# Cell landscape of larval and adult *Xenopus laevis* at single-cell resolution

**DOI:** 10.1038/s41467-022-31949-2

**Published:** 2022-07-25

**Authors:** Yuan Liao, Lifeng Ma, Qile Guo, Weigao E, Xing Fang, Lei Yang, Fanwei Ruan, Jingjing Wang, Peijing Zhang, Zhongyi Sun, Haide Chen, Zhongliang Lin, Xueyi Wang, Xinru Wang, Huiyu Sun, Xiunan Fang, Yincong Zhou, Ming Chen, Wanhua Shen, Guoji Guo, Xiaoping Han

**Affiliations:** 1 Center for Stem Cell and Regenerative Medicine, and Bone Marrow Transplantation Center of the First Affiliated Hospital, Zhejiang University School of Medicine, Hangzhou, 310000 China; 2 Zhejiang Provincial Key Lab for Tissue Engineering and Regenerative Medicine, Dr. Li Dak Sum & Yip Yio Chin Center for Stem Cell and Regenerative Medicine, Hangzhou, Zhejiang 310058 China; 3 ZJU-UOE Institute, Zhejiang University, School of Medicine, Haining, Zhejiang China; 4 Liangzhu Laboratory, Zhejiang University Medical Center, Hangzhou, Zhejiang 311121 China; 5 College of Life Sciences, Zhejiang University, Hangzhou, 310003 China; 6 Zhejiang Key Laboratory of Organ Development and Regeneration, College of Life and Environmental Sciences, Hangzhou Normal University, Hangzhou, Zhejiang China

**Keywords:** RNA sequencing, Developmental biology, Biological models, Gene expression analysis

## Abstract

The rapid development of high-throughput single-cell RNA sequencing technology offers a good opportunity to dissect cell heterogeneity of animals. A large number of organism-wide single-cell atlases have been constructed for vertebrates such as *Homo sapiens*, *Macaca fascicularis*, *Mus musculus* and *Danio rerio*. However, an intermediate taxon that links mammals to vertebrates of more ancient origin is still lacking. Here, we construct the first *Xenopus* cell landscape to date, including larval and adult organs. Common cell lineage-specific transcription factors have been identified in vertebrates, including fish, amphibians and mammals. The comparison of larval and adult erythrocytes identifies stage-specific hemoglobin subtypes, as well as a common type of cluster containing both larval and adult hemoglobin, mainly at NF59. In addition, cell lineages originating from all three layers exhibits both antigen processing and presentation during metamorphosis, indicating a common regulatory mechanism during metamorphosis. Overall, our study provides a large-scale resource for research on *Xenopus* metamorphosis and adult organs.

## Abstract (web summary)

Single-cell RNA sequencing technology offers a unique opportunity to dissect cell heterogeneity of animals. Here, the authors construct a Xenopus cell landscape including larval and adult organs to dissect cell heterogeneity of the amphibian.

## Introduction

The rapid development of high-throughput single-cell RNA sequencing (scRNA-seq) technology offer a good opportunity to dissect cell heterogeneity of *Xenopus*, from embryogenesis^1,2^ to individual organs such as cornea and kidney^3,4^, and to reveal the cellular mechanism of *Xenopus* regeneration such as in limb^5,6^, tail^7,8^ and spinal cord^9^. A large number of organism-wide single-cell atlases have been constructed for various species, such as *Homo sapiens*^10^, *Macaca fascicularis*^11^, *Mus musculus*^12^, *Danio rerio*^13^, *Drosophila melanogaster*^14^, *Ciona intestinalis*^15^, *Caenorhabditis elegans*^16^, *Schmidtea mediterranea*^17^, and *Stylophora pistillata*^18^. From an evolutionary perspective, the amphibian is an intermediate taxon that links mammals to vertebrates of more ancient origin. A comprehensive larval and adult *Xenopus* cell landscape is helpful in all aspects of a better understanding of genetic evolution relations. For example, to achieve the transition from an aquatic lifestyle to a terrestrial one, anuran tadpoles undergo drastic morphological and physiological changes during metamorphosis, such as the appearance of limbs, loss of larval tail, remodeling of digestive organs, and decrease in regeneration ability^19^. The mammalian perinatal period shares strong similarities with amphibian metamorphosis^20^, making the process of metamorphosis an excellent model from which to learn about mammalian postembryonic development. However, the molecular networks that regulate metamorphosis at single-cell resolution are still unclear.

In this work, we perform highly parallel single-cell transcriptomics with Microwell-seq to construct an initial “*Xenopus* Cell Landscape (XCL)”, comprising more than 500,000 cells isolated from four *Xenopus laevis* stages, from larva to juvenile, and 17 adult tissues. XCL, the first such comprehensive resource for clawed frog research, profiles more than 100 major cell types in *Xenopus laevis*, which provides valuable scientific evidence of cross-species evolution. The XCL database website is publicly available at http://bis.zju.edu.cn/XCL/ for all researchers in the *Xenopus* community.

## Results

### Construction of the *Xenopus* cell landscape using Microwell-seq

**Fig. 1:**
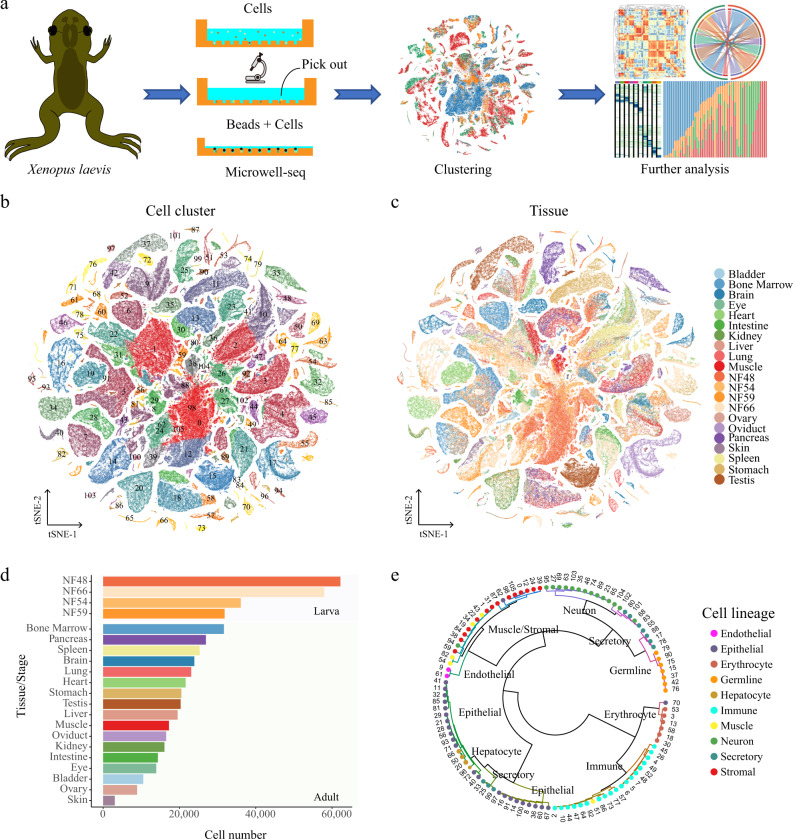
Constructing an XCL using Microwell-seq. **a** A schematic of the basic workflow for the *Xenopus* cell landscape using the Microwell-seq platform. **b** t-SNE analysis of 501, 358 single cells collected from larval and adult tissues. The 106 cell-type clusters are labeled in different colors. Cell cluster markers are listed in Supplementary Dataset 1. **c** t-SNE analysis of 501, 358 single cells collected from larval and adult tissues. Tissues/stages are labeled in different colors. **d** Cell number of each tissue/stage is detected at the XCL. Tissues/stages are labeled in different colors. **e** The 106 cell-type clusters are re-clustered into ten cell lineages and circles showing the relationships among the 106 cell types. Cell lineages are labeled in different colors.

*Xenopus laevis* is a classical model of vertebrate embryonic development^1^, regeneration potential^8^, and neuronal regulation^21^. Using Microwell-seq, we constructed a comprehensive XCL including adult and larval metamorphosis stages (Fig. 1a)^12^. A detailed multi-organ transcriptome landscape covered brain, liver, lung, kidney, intestine, heart, muscle, skin, pancreas, eye, testis, ovary, bladder, stomach, bone marrow, spleen, and oviduct tissues from 1-year-old *Xenopus laevis*. The whole body of Nieuwkoop and Faber (NF) stage 48 (premetamorphosis), NF54 (prometamorphosis), NF59 (metamorphosis climax), and NF66 (end of metamorphosis) were dissociated respectively to fully analyze the process of metamorphosis. Tissues and tadpoles were prepared as single-cell suspensions (Supplementary Dataset 1).

The single-cell transcriptomics data were processed using published pipelines^22^. In total, we obtained 501, 358 qualified single cells from 17 adult tissues and tadpoles at NF48, NF54, NF59, and NF66 (Fig. 1b–d and Fig. S1a, b). The complete tissue dataset was grouped into 106 clusters and over 581 cell-type subclusters in the hierarchy (Fig. 1b, c and Fig. S1c). The numbers of cells and unique molecular identifiers (UMIs) per cell detected for each tissue are shown in Fig. 1d and Fig. S1b. The average number of genes detected per cell was ~600. The tissues with the highest and lowest average numbers of genes detected were the testis and pancreas, with ~1000 genes and 300 genes respectively (Fig. S1a). We annotated the cell types based on clustering markers referred in previous studies. The marker genes for each cell type are listed in Fig. S1d and Supplementary Dataset 1. Multiple tissues, including bladder, eye, heart, lung, muscle, ovary, pancreas, oviduct, and stomach, contributed to the defined adult stromal cells (Cluster (C)1, C22, and C59), while several clusters (C0, C12, C24, and C105) were larva-specific stromal cells. Other clusters with significant multi-tissue contributions corresponded to epithelial cells (C8), endothelial cells (C9), and muscle cells (C43) (Fig. 1b, c).

To define the cell-type similarities and relationships, pairwise unsupervised MetaNeighbor^23^ analyses were performed for these 106 clusters (Fig. 1e). The resulting circular cell-type dendrogram was organized according to major lineages rather than tissues. For example, most cell types were matched within the same category, such as stromal, muscle, and epithelial cells.

In total, our landscape provided cellular signatures for major tissues of *Xenopus laevis*, providing a valuable resource at single-cell resolution for the *Xenopus* community. The resource is publicly available at http://bis.zju.edu.cn/XCL/.

### Cellular heterogeneity in diverse *Xenopus laevis* tissues

**Fig. 2:**
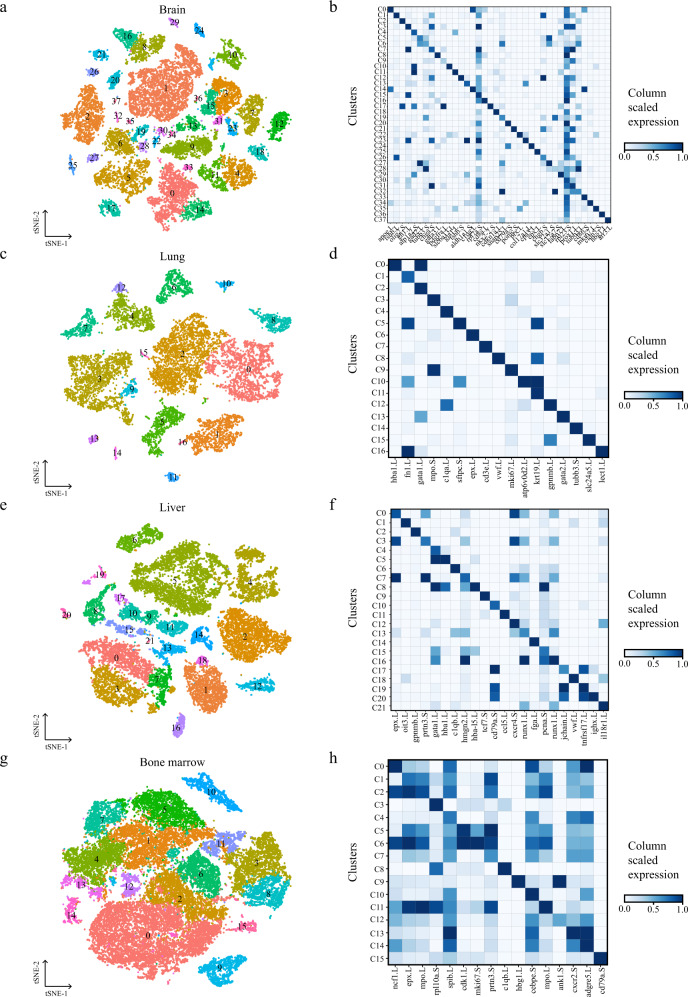
Cellular heterogeneity in diverse adult *Xenopus laevis* tissues. t-SNE map of *Xenopus* brain (**a**), lung (**c**), liver (**e**), and bone marrow (**g**) single-cell data. Cells are colored by cell-type cluster; Heatmap showing representative gene expression in each cluster of *Xenopus* brain (**b**), lung (**d**), liver (**f**), and bone marrow (**h**). The color encodes the average expression level.

Using t-distributed stochastic neighbor embedding (t-SNE) and differential gene expression analyses, we constructed more detailed transcriptional profiles of *Xenopus* tissues, including previously unrecognized cell heterogeneity. The brain is the most complicated tissue, and our data covered most known cell types, such as excitatory and inhibitory neurons, radial glia, astrocytes and oligodendrocytes. We also identified several endocrine cells from the pituitary gland, including gonadotroph cells, growth hormone cells, thyrotroph cells, and melanotrope cells (Fig. 2a and Supplementary Dataset 2). We further examined specific marker genes related to each cluster. Three GABAergic neurons (inhibitory neuron) subclusters (C5, C6, and C27) expressed high levels of *slc32a1* and *gad2*, whereas three excitatory neuron subclusters (C19, C28, and C32) expressed high levels of *slc17a7*. Myelinating oligodendrocytes (C2) expressed high levels of myelin-associated genes *plp1*, *mbp,* and *olig2*, and progenitor cells (C17) expressed high levels of *pdgfra* and the neurogenesis-related transcription factor *nkx2-2*^24^ (Fig. 2b and Supplementary Dataset 2). Notably, a large number of endocrine cells were identified from the pituitary gland that expressed different hormone-associated genes as well as co-expression of high levels of *chga* and *chgb* (Fig. S2). For instance, gonadotroph cells (C1 and C26) expressed high levels of *fshb*, growth hormone cells (C3, C7, and C15) expressed high levels of *gh1*, prolactin progenitor cells (C9) expressed high levels of *prl.1*, and thyrotroph cells (C12) expressed high levels of *tshb*. In addition, C10, which expressed the astrocyte marker *atp1a2*, was identified as representative fibrous astrocytes for its high expression of the myelin-associated gene *pmp2*^25^ (Fig. 2b).

The lung is an organ of particular interest because *Xenopus* undergoes a transition from living in an aquatic environment to living in a terrestrial environment. We annotated 17 cell-type clusters in the lung with analysis based on known markers (Fig. 2c, d and Supplementary Dataset 2). Alveolar epithelial (AE) cells (C5) were enriched in *sftpb* and *sftpc*, which are typical markers of alveolar type 2 (AT2) cells in the mouse and human lungs (Fig. S3a). The lack of alveolar type 1 (AT1) cells which are easily found in the lungs of mice and humans were missed in our data and enriched pulmonary ionocytes (C10) with high expression of *atp6v0d2* and *atp6v1b1* (Fig. 2d and Fig. S3a), which implied that the respiratory organ of *Xenopus* was intermediate between the gill enrichment of ionocytes and mammalian lung enrichment of alveolar epithelial cells. We also identified melanocytes (C15) with high expression of *pmel-like* and *tyrp1*, which were previously reported to exist in the lungs of *Xenopus* with unknown function^26^ (Fig. 2c and Supplementary Dataset 2). To infer the evolutionary relationship between the mouse lung and zebrafish respiratory organs, we performed a cross-species clustering analysis of these tissues. To enable cross-species analysis, orthologous and paralogous genes from mouse, zebrafish and *Xenopus* were extracted^27^. As revealed in Fig. S3b, several cell types were conserved among zebrafish, *Xenopus*, and mouse. For example, the gene expression patterns of *Xenopus* pulmonary ionocytes showed strong correlations with zebrafish gill ionocytes, zebrafish swim bladder epithelial cells and mouse AT2 cells. Interestingly, although *sftpb* and *sftpc* were highly expressed, the gene expression patterns of *Xenopus* AE cells showed strong correlations with not only mouse AT2 cells but also mouse AT1 cells, Club cells, and alveolar bipotent progenitors, indicating that *Xenopus* AE cells performed a variety of functions similar to those of mouse AT1, AT2 and Club cells in adult *Xenopus* lungs. *Xenopus* AE cells also showed strong correlations with zebrafish swim bladder epithelial cells, implying the evolutionary relationships among the *Xenopus* lung, zebrafish swim bladder, and mouse lung. These results were consistent with a previous hypothesis that cell types rather than organs might be proposed as “evolutionary units” in evolutionary biology^28,29^.

Unlike that in mammals, hematopoiesis in *Xenopus* occurs in the liver rather than bone marrow^30,31^. To further study the function of hematopoiesis at the single-cell level, we analyzed the cellular components in *Xenopus* liver and bone marrow, respectively. We identified 21 clusters in the *Xenopus* liver (Fig. 2e, f) and expectedly most were hematopoiesis-related clusters except liver parenchyma cell hepatocytes (C14). For example, hematopoietic stem and progenitor cells (HSPCs and C16) were enriched in hematopoiesis transcription factors *gata2*, *runx1*, and *tal1* (Fig. 2f and Supplementary Dataset 2). Next, we identified erythrocytes at various differentiation stages, including erythroid progenitor cells (C4), proliferating early erythrocytes (C15), proliferating middle erythrocytes (C8), and late erythrocytes (C5) based on previously reported marker genes^32^ (Fig. S4a). In addition, among 16 identified clusters in *Xenopus* bone marrow, mainly composing granulocytes such as neutrophils and eosinophils (Fig. 2g, h). In contrast to adult mouse bone marrow, *Xenopus* bone marrow contained no HSPCs, but its other cellular components, such as major granulocytes, were similar (Fig. S4b). These data indicated that the liver was the primary hematopoietic organ of adult *Xenopus* while the bone marrow harbored granulocytic potential.

Further, we defined cell-type clusters and their markers in all the other tissues and summarized them in Supplementary Dataset 2 and on the XCL website (Fig. S5 and Supplementary Dataset 2).

### Cell-type evolution between adult *Xenopus*, zebrafish, and mammals

**Fig. 3:**
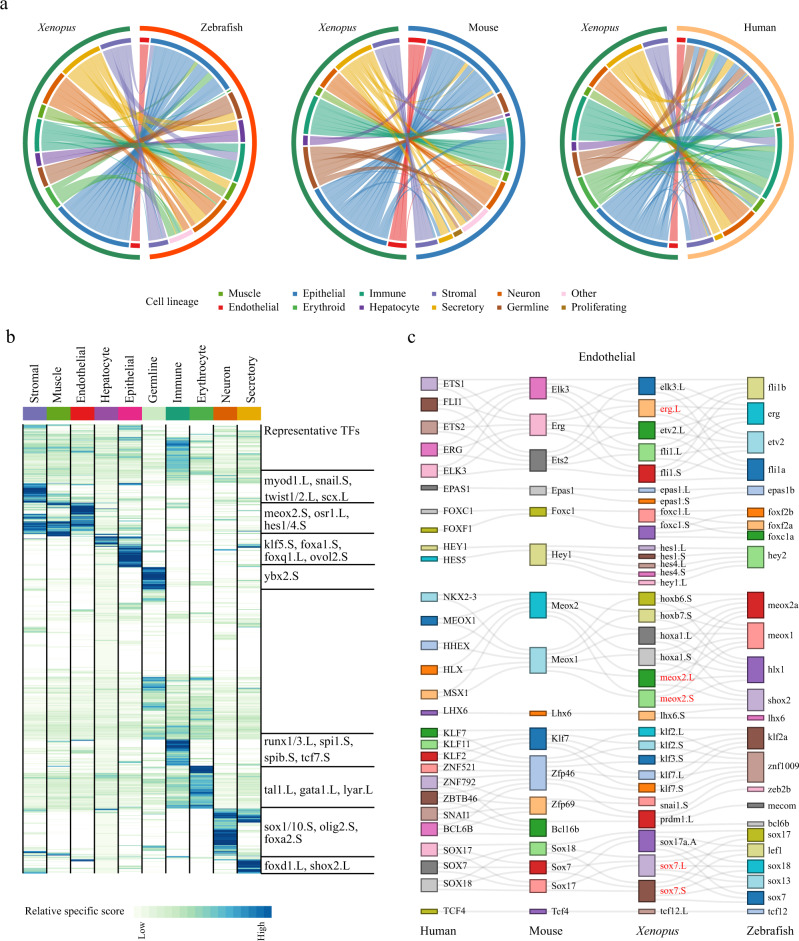
Cell-type evolution between adult *Xenopus*, zebrafish, and mammals. **a** Circle plot showing the similarity of cell lineages in *Xenopus laevis*, zebrafish (left), mice (middle), and humans (right). Pairs of cell types with mapping scores greater than 0.1 are connected by lines. Cell lineages are labeled in different colors. **b** Heatmap showing the specific scores of TFs in *Xenopus* for each cell type. Each row represents one TF, and each column represents one cell lineage. Cell lineages are labeled in different colors. The representative TFs in each cell lineage are presented in the right panel. **c** Sankey plot showing common lineage-specific transcription factors in endothelial for four vertebrates (humans, mice, *Xenopus*, and zebrafish). The representative TFs in *Xenopus laevis* are labeled by red. Different TFs in each species are marked in different color boxes. Homologous TFs between species obtained from SAMap are connected by lines.

Cross-species single-cell RNA analysis provides new insight into animal evolution at the single-cell level^28,33^. To identify the evolutionary process in vertebrates, updated datasets from the Human Cell Landscape (HCL), the Mouse Cell Atlas (MCA), and the Zebrafish Cell Landscape (ZCL) were collected. Details of selected homologous genes across four species were shown in Supplementary Dataset 3. To proceed with network construction, pseudocells for each cell cluster of *Xenopus laevis* were constructed and an integrated manifold with a high degree of cross-species alignment was produced by SAMap to ensure the accuracy of cell types and cell states comparison (Fig. S6). As shown in Fig. 3a, the gene expression patterns of endothelial cells showed the strongest correlations between both species compared with other lineages. Besides, stromal cells were also highly conserved across these four species. The germline of *Xenopus* showed the strongest correlation with the zebrafish germline and lower correlations with mouse germline, and few correlations with human germline, indicating that the germline of *Xenopus* was evolutionarily closer to fish than to mammals.

To investigate the genetic network that underlies cell lineage fate determination, transcription factor specificity scores were calculated for each cell type of *Xenopus laevis* (Fig. 3b). Stromal, muscle, and endothelial cells shared several TFs, such as the *notch* effector *hes1/4*, which are reported to regulate myogenesis^34,35^. *Twist1/2* and *snail*, which are related to the epithelial-to-mesenchymal transition in humans, were highly associated with stromal cells^36^. *Meox2*, a response gene for cell migration and fatty acid transport, was identified in endothelial cells^37,38^. The key regulators of erythropoiesis *gata1* and *tal1* in mice were also highly associated with erythrocytes in *Xenopus*^39,40^. For immune cells of *Xenopus*, the major hematopoietic TF *runx1* and the key regulator of T cell differentiation *runx3* were identified^41,42^. *Sox1*, reported to be implicated in *Xenopus* neurogenesis and the *Xenopus* myelination-related gene *sox10* were detected in our neuronal cells^43,44^ (Fig. 3b). To confirm the conserved cell-lineage-specific TFs in vertebrates, we collected TF data from humans, mice, and zebrafish, and further identified conserved TFs for each lineage based on homologous relationships and expression (Fig. 3c and Figs. S7, S8). Our data showed that endothelial cells shared several TFs in all lineages, such as the regulator of endothelial differentiation *erg*^45^, a key gene for vascular development *sox7*^46^, and the regulator of transcription of lipid transport *meox2* for endothelial cells^47^ (Fig. 3c). In addition, neurons shared most TFs in all lineages. For instance, the key neural crest specifier *sox10*^48^, a common regulator of the neuronal conversion gene *neurod1*^49^, and the regulator of the oligodendrocyte lineage and neural cell specification *olig2* for neurons^50^ (Fig. S8a). In particular, some highly conserved TFs such as the regulator of neural stem cells *tcf4*^51^ and the known neural developmental regulator *lhx6*^52^ were detected in four species (Fig. S8a). Notably, *olig1*, a common oligodendrocyte transcription factor in mice and humans^53^, was not detected in *Xenopus* adult neurons and was only weakly expressed prior to the tadpole stage^54^ (Fig. S8a).

### Cell landscape of larval *Xenopus* during metamorphosis

**Fig. 4:**
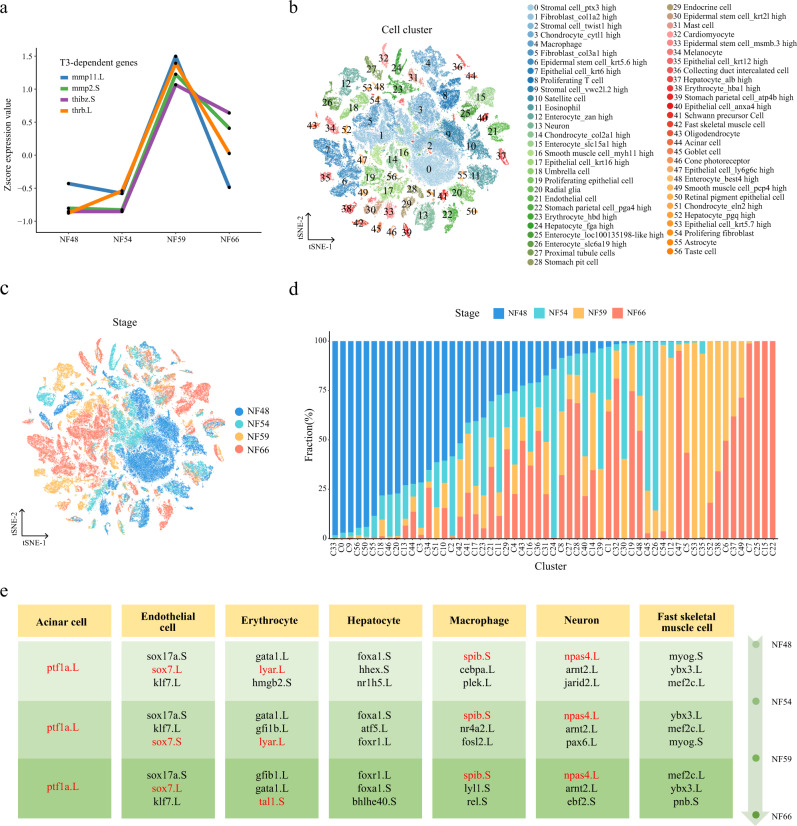
Cell landscape of larval *Xenopus* during metamorphosis. **a** Expression trajectory of genes regulated by T3 during metamorphosis. **b**, t-SNE analysis of 188, 020 single cells collected from larval *Xenopus*. 57 cell-type clusters are labeled in different colors. Cell cluster markers are listed in Supplementary Dataset 1. **c** t-SNE analysis of 188, 020 single cells collected from larval *Xenopus*. Stages are labeled in different colors. **d** Stage proportion in different cell clusters. Stages are labeled in different colors. **e** dTFs are listed in different stages for cell lineages. Each row represents one stage and each column represents one cell type. The representative TFs for each cell types are labeled by red.

To describe the cellular mechanisms regulating amphibian metamorphosis at the single-cell level, NF48, NF54, NF59, and NF66 tadpoles were processed using Microwell-seq and 188,020 single cells passed our quality control tests. Metamorphosis is a process regulated by thyroid hormone (TH) T3. Previous research indicates that T3 is able to induce complete metamorphosis and blockade of T3 synthesis prevents metamorphosis^55^. To ensure that the collected samples represented different stages of metamorphosis, we cross-referenced our data with the expression patterns of four previously reported T3-dependent genes^56^, which indicated that our samples represented premetamorphosis, prometamorphosis, metamorphic climax, and the end of metamorphosis (Fig. 4a). The marker genes for each major cell type were listed in Fig. S9a. In our data, several major cell type categories were present in all four stages, such as the defined neurons (C13, C20, C29, C41, and C43), immune cells (C4, C11, and C31), and proximal tubule cells (C27) (Fig. 4b, c). Several digestion system clusters were specifically present in certain stages. For instance, stomach parietal cells (C22) and hepatocytes (C24) were present only in NF66 and NF54, respectively. Meanwhile, enterocytes (C12) were present in NF59 and enterocytes (C15 and C25) were present in N66; however, enterocytes (C48) were present in NF54, NF59, and NF66. Our results showed that remodeling of the intestine is the one of the most dramatic changes during metamorphosis as noted previously^57^. Enterocytes, especially at later stages, exhibited high heterogeneity due to changes in diet during metamorphosis to form the complex adult intestine^56,58^.

Next, the different stage tadpole datasets were grouped into 38, 46, 48, and 56 clusters respectively (Fig. S9b–e). We analyzed the proportions of four stages in each cluster. As shown in Fig. 4d, almost no intertemporal cell types emerged, indicating reliable temporality of our data. To further understand the transformations that occur during metamorphosis, we performed another round of clustering specifically for epithelial, stromal, and immune cells (Fig. S10 and Supplementary Dataset 4). Obviously, epithelial and stromal cells exerted strong transcriptomic heterogeneity rather than immune cells. Overall, NF66 is more self-consistent in Epithelial and Stromal in batch-integrated cellular profiles. We also analyzed the relationship between larval and adult cell clusters (Figs. S11–S12 and Supplementary Dataset 5). As shown in Fig. S11b, c, the enterocytes at NF66 and adult shared transcriptional similarity, while enterocytes at NF54 and NF59 show stronger heterogeneity. The correlation of clusters and DGEs between larval and adult enterocytes were shown in Fig. S11c. The high correlation coefficient between C3 and C4 with the most proportion of NF54 and NF59 data suggested some NF-specific regulation, while DGEs contributing to the combined cluster from larval and adult integration indicated that enterocytes were undergoing extensive remodeling during metamorphosis. Similar to the enterocytes, most stomach parietal cells at NF66 and adults shared transcriptional similarity but low correlation with that at NF54 and NF59 (Fig. S11d–f). Hepatocytes at NF54 showed transcriptional heterogeneity but hepatocytes at NF59 displayed a high correlation with NF66 and adult hepatocytes (Fig. S12a–c). In terms of neuron cells, general integration showed a high correlation among adult and most larval neurons, with some adult neurons showing heterogeneity mostly in C1 and C3 (Fig. S12d–f). Taken together, these data suggested that significant transcriptome changes occurred in the vast majority of adult cells compared with larval cells that underwent metamorphosis.

To identify the stage-specific TFs for each cell type, we collected TF data from all cell types at four stages and defined the stage-specific TFs as “driver TFs (dTFs)” (details in methods and Supplementary Dataset 6). As shown in Fig. 4e, several cell types, including endothelial cells and acinar cells, exhibited relatively stable regulatory factor expression during metamorphosis. By contrary, erythrocytes dTFs underwent tremendous changes, with the exception of *gata1*. It was worth noting that *lyar*, which regulates globin gene expression during development was no longer a specific transcription factor at NF66^59^, indicating a hemoglobin (Hb) transition during this stage. Meanwhile, *tal1*, a regulator of erythroid differentiation, became a specific transcription factor at NF66^60^. These changes were likely important for the larval erythrocytes to undergo Hb transition and to differentiate into adult erythrocytes. In total, our data provided a useful resource for identifying the key regulators of each cell lineage during metamorphosis.

### Conversion of erythrocyte hemoglobin from larval to adult

**Fig. 5:**
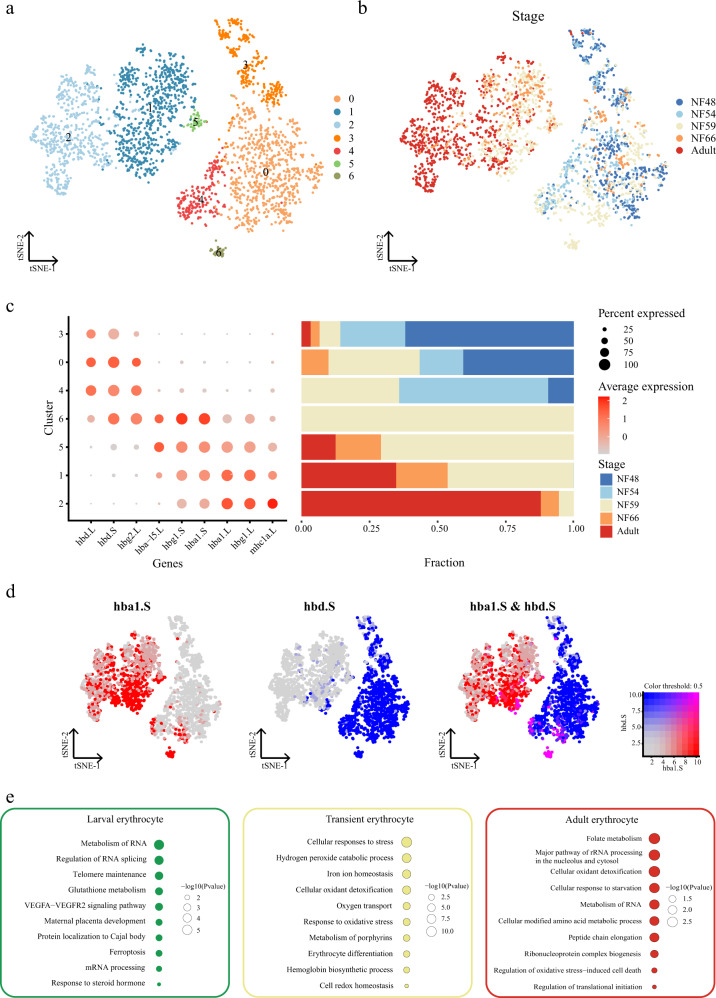
Conversion of erythrocyte hemoglobin from larva to adult. **a** t-SNE analysis of erythrocytes collected from larval and adult *Xenopus*. Different clusters are labeled in different colors. Erythrocytes from adults were sampled to the same order of magnitude as in larva. Cell cluster markers are listed in Supplementary Dataset 3. **b** t-SNE analysis of erythrocytes collected from larval and adult *Xenopus*. Different stages are labeled in different colors. **c** Representative gene expression in each cluster of erythrocytes and cell proportions in different stages are shown. Stages are labeled in different colors. The size of the dot encodes the percentage of cells within a cell type, and the color encodes the average expression level. **d** Feature plot in the t-SNE map of erythrocytes collected from larval and adult *Xenopus*. Cells are colored according to the expression of the indicated marker genes *hba1.S* (red), *hbd.S* (blue), and co-expression *hba1.S* and *hbd.S* (pink). **e** Representative GO terms enriched in each module for three cell types. Different cell clusters are labeled in different colors. *P* value was calculated by the hypergeometric distribution, a statistical test is one-sided, adjustments *P* values were made after *P* value is corrected by Benjamin–Hochberg multiple test.

Hb transition from larva to adult during vertebrate development is a physiologically important process for animals to adapt to environmental changes from the fetal environment to an atmospheric environment in animals. Hb switching is also TH-dependent and which kinds of globin are expressed in individual erythrocytes at different stages in *Xenopus laevis* has been controversial^61^. To investigate this transition during metamorphosis in *Xenopus*, we reanalyzed the whole larval and adult erythrocytes. As expected, adult and larval erythrocytes showed strong heterogeneity before and after metamorphosis (Fig. 5a, b). In adults and NF66, erythrocytes mainly expressed *hba1* and *hbg1*, while in early tadpoles at NF48 and NF54, erythrocytes expressed *hbg2* and *hbd* instead. Notably, during premetamorphosis at NF59, the cluster of erythrocytes coexpressing *hba1*, *hbg1*, *hbg2*, and *hbd* indicated a transient state in the development of erythrocytes (Fig. 5c). The expression of *hba1* and *hbd* confirmed that hemoglobin was co-expressed in individual erythrocytes (Fig. 5d). Although several reports have claimed that no erythrocytes contained both larval and adult globin^61,62^, our data identified that there was a cluster of erythrocytes, mainly at NF59, that contained both larval and adult globin, as reported previously^63,64^. Interestingly, our data showed that few or no *mhc1a* (MHC class I) molecules were detected in larval erythrocytes before metamorphosis, consistent with a previous report (Fig. 5c), but a continuous increase in expression occurred following development until adult erythrocyte transition^65^. Our result suggested that immune cells might be involved in the removal of larval erythrocytes without *mhc1a*. To investigate the molecular pathways involved in erythrocyte development, we sub-clustered whole erythrocytes into three groups: a larval cluster (C0) of erythrocytes mainly at NF48 and NF54; a transient cluster (C6) of erythrocytes at NF59; and an adult cluster (C2), of erythrocytes mainly at NF66 and in adults. GO classification was performed to analyze the biological functions of the differentially expressed genes (DEGs) in each group based on human homologs (Fig. 5e). The larval cluster was enriched mainly in biological pathway categories associated with RNA metabolism and telomere maintenance, suggesting that transcription and telomerase activity were necessary at the early stage of erythrocytes. Erythrocyte differentiation- and hemoglobin biosynthesis-related biological pathways were enriched in transient clusters. This result implied that erythrocytes at this stage might synthesize new hemoglobin and differentiate into new cell types during the transient state. In conclusion, our data indicated that *hba1* and *hbg1* are adult-special hemoglobin in *Xenopus laevis*, while *hbg2* and *hbd* are larval-special hemoglobin, and that there is a transient cluster of cells containing both larval and adult hemoglobin only at NF59 that might be a progenitor of adult erythrocytes.

### Gene expression profiles of the remodeling multisystem during metamorphosis

**Fig. 6:**
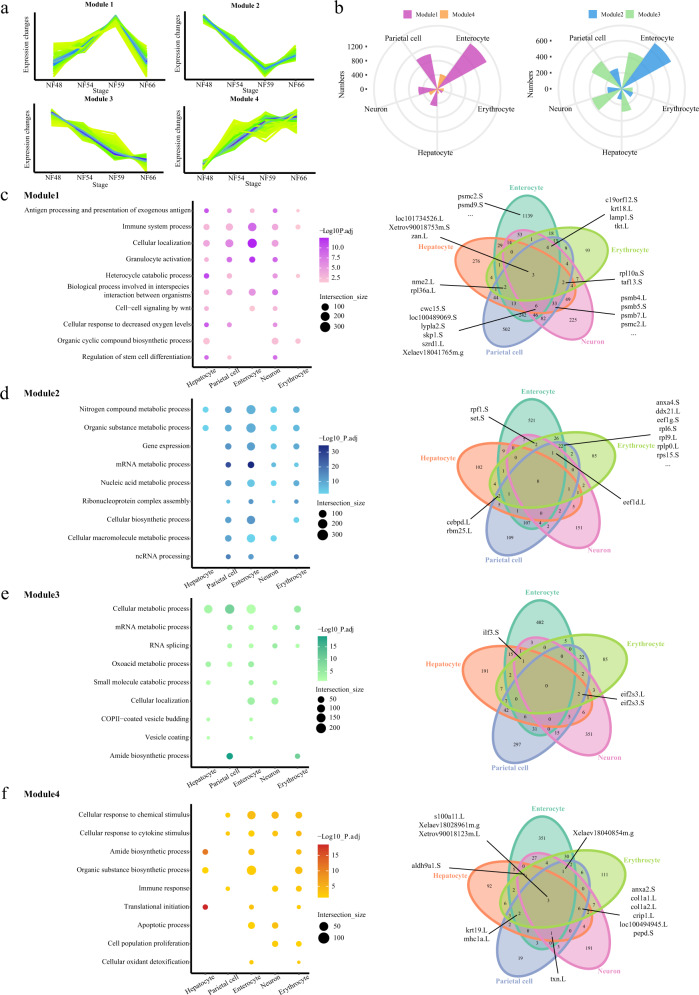
Gene expression profiles of the remodeling multisystem during metamorphosis. **a** Schematic diagram of the four modules. **b** Rose diagrams showing the distribution of DEGs in each cell type. Different modules are labeled in different colors. **c**–**f** Representative GO terms enriched in module 1 (**c**), 2 (**d**), 3 (**e**), and 4 (**f**) for five cell types, respectively (left). GO terms enriched in different modules are labeled in different colors. Venn diagrams showing the numbers of shared genes in each module (right). The bold lines indicate the representative genes shared in at least three cell types. *P* value was calculated by the hypergeometric distribution, statistical test is one-sided, adjustments *P* values were made after *P* value is corrected by Benjamin–Hochberg multiple tests.

As shown in Fig. 4c, digestion system cells derived from the endoderm, including enterocytes, hepatocytes and stomach parietal cells showed strong heterogeneity during metamorphosis. To investigate common molecular pathways involved in tissue development during metamorphosis, we re-collected data from digestion system cells, erythrocytes derived from mesoderm and neurons derived from ectoderm to analyze. We clustered selected genes expressed (for details, see Methods) into four modules: module 1, thyroid hormone-synchronous upregulated genes; module 2, thyroid hormone-synchronous downregulated genes; module 3, continuously downregulated genes; module 4, continuously upregulated genes (Fig. 6a and Supplementary Dataset 7). The number of genes in module 1 was larger than that of module 4 for each cell lineage (Fig. 6b), indicating that upregulated genes involved in metamorphosis were mainly thyroid hormone-dependent.

Next, GO analysis was performed (based on the human homologs) to clarify the biological and molecular functions of the genes in each module (Fig. 6c–f). The entire GO term for each module could be found in Supplementary Dataset 8. The common genes in module 1 across cell types during metamorphosis were enriched in antigen processing and presentation of exogenous antigen, immune system process, cellular location, granulocyte activation, and heterocycle catabolic process. Interestingly, the shared genes enriched in antigen processing and presentation of exogenous antigen catalog were proteasome family genes, although different subtypes, such as *psmb4*, *psmb5*, and *psmc2*, are involved in the expression of MHC class I antigens and protein degradation^66^. MHC class I antigens were reported to be important surface markers of adult erythrocytes during *Xenopus* metamorphosis^67^. Our data suggested that antigen processing and presentation in NF59 was required for the formation of MHC I molecules at the end of metamorphosis and was a common regulatory mechanism in each lineage during metamorphosis. To detect the common genes shared by multiple cell types, we also generated Venn diagrams of the genes in different cell types. Genes present with a high frequency in module 1 were *zan* (freq = 5), *nme2* (freq = 4), *cwcl5* (freq = 4), *lypla2* (freq = 4), *taf13* (freq = 4), and so on.

The common genes in module 2 across cell types during metamorphosis were mainly enriched in metabolic processes such as nitrogen compound metabolic process, organic substance metabolic process, mRNA metabolic process, and cellular macromolecule metabolic process. The common genes in module 3 across cell types during metamorphosis were also mainly enriched in metabolic processes, such as cellular metabolic process, mRNA metabolic process, and oxoacid metabolic process (Fig. 6d, e). These data suggested that some cellular metabolic processes such as nitrogen compound and mRNA metabolic process were continually downregulated from the climax of metamorphosis to the end of metamorphosis. Genes with a high frequency in module 2 and 3 were also mainly translation-related genes, such as *eef1d* (freq = 4), *eif2s3* (freq = 4), and *ilf3* (freq = 4), which are involved in the elongation and translation initiation process of protein synthesis^68,69^.

The common genes in module 4 across cell types during metamorphosis were enriched in cellular response to chemical or cytokine stimulus, amide biosynthetic process, immune response and translational initiation. These data suggested that upregulation of these processes at the end of metamorphosis was required for the maturation of the adult organ. Genes with a high frequency of module 4 were *s100a11* (freq = 5), *aldh9a1* (freq = 4), *anxa2* (freq = 4), *col1a1* (freq = 4), *col1a2* (freq = 4), *crip1* (freq = 4), *krt19* (freq = 4), *mhc1a* (freq = 4), *pepd* (freq = 4), and *txn* (freq = 4). Notably, these genes are involved in diverse cellular processes, highlighting important biomarkers and targets for metamorphosis in *Xenopus*.

Overall, our data demonstrated that upregulation of antigen processing and presentation and downregulation of cellular metabolic processes are common biological processes in *Xenopus* metamorphosis for major cell lineage.

## Discussion

The constructed organism-wide cell atlases, such as those for *Homo sapiens*, *Mus musculus*, and *Danio rerio*, alongside that of *Xenopus laevis* generated here, allowed us to systematically compare cell types across vertebrates. During the transition from an aquatic to a terrestrial lifestyle, the respiratory system of amphibians undergoes drastic changes. The evolutionary relationships among the lung, gill and swim bladder are controversial based on current evidence^70^. Our data identified that *Xenopus* AE cells showed strong correlations with zebrafish swim bladder epithelial cells and mouse AT2 cells, AT1 cells, Club cells and alveolar bipotent progenitors. Meanwhile, *Xenopus* pulmonary ionocytes showed strong relationships to zebrafish gill ionocytes, zebrafish swim bladder epithelial cells, and mouse AT2 cells. Recently, cell types rather than tissues have been proposed as “evolutionary units”^28,29^. Our results also demonstrated that the evolutionary conservation and divergence of the vertebrate respiratory system relied mainly on cell types and not tissues, as previous research proposed.

Amphibian metamorphosis is a dramatic biological phenomenon that could be controlled by the simple TH. Our data indicated that antigen processing and presentation was a common regulatory mechanism involved in cell apoptosis in lineages derived from three germ layers during metamorphosis. GO analysis of thyroid hormone-synchronous upregulated genes during metamorphosis revealed enrichment in genes associated with antigen processing and presentation of exogenous antigens. MHC class I antigens were used to distinguish larval and adult erythrocyte clusters in a previous study^67^. The absence of MHC antigens on the surface of larval erythrocytes is consistent with our results. However, larval erythrocytes were cleared from circulation after 60 days after metamorphosis, indicating a mechanism for the protection of adult erythrocytes after metamorphosis. To our surprise, other cell types, such as hepatocytes, enterocytes, neurons, and stomach parietal cells, contained both antigen processing and presentation genes. These data from ectoderm, mesoderm, and endoderm implied that antigen processing and presentation might be a shared biological pathway involving tissue remodeling during metamorphosis.

In conclusion, we constructed the first *Xenopus* cell landscape at the single-cell level, providing large-scale resource for research on *Xenopus* metamorphosis and adult organs. We performed comparative analyses among vertebrate species at single-cell resolution and revealed an evolutionary relationship between the respiratory systems of aquatic and terrestrial animals. We identified a common biological pathway involving cell apoptosis and differentiation for cell lineages from the three germ layers. These data are valuable for future research in vertebrate biology.

## Methods

### Animals

Stage 48–66 tadpoles (ten in NF48 and NF54 and one in NF59 and NF66) were obtained from lab-bred mature *Xenopus laevis* (from Shen’s lab at Hangzhou Normal University) and reared in a 22 °C incubator with 12 h light /12 h dark cycle in 0.1×Steinberg’s solution [in mM: 10 HEPES (Cat. No. H3375, Sigma), 58 NaCl (Cat. No. S5886, Sigma), 0.67 KCl (Cat. No. P5405, Sigma), 0.34 Ca(NO_3_)_2_ (Cat. No. 31218, Sigma), 0.83 MgSO_4_ (Cat. No. M2643, Sigma), pH 7.2]. The adult *Xenopus laevis* (wide type, including three males and two females) aged from 1-year older were studied. All animal protocols in this study were approved by the Ethics Committee of the Zhejiang University Laboratory Animal Center (Lot number: 20201049 and ZJU20220195).

### Fabrication of the microwell device

Firstly, a silicon plate (Suzhou chip scientific instrument Co., Ltd., Suzhou, China) containing 100,000 microwells whose diameter and depth were 28 and 35 µm respectively was manufactured. Secondly, a polydimethylsiloxane (PDMS) plate with the same number of micropillars was made using the silicon microwell plate. Lastly, the PDMS plate was used as a mold to make a disposable agarose microwell plate by pouring 5% agarose (Cat. No. BY-R0100, Baygene) solution onto the surface of the PDMS plate.

### Synthesis of barcoded beads

For bead synthesis, 350 µl carboxyl magnetic beads (20–25 µm in diameter, 50 mg/ml, Suzhou Knowledge & Benefit Sphere Tech. Co., Ltd., Suzhou, China) were washed twice with 0.1 M 2-[N-morpholino] ethanesulfonic acid (MES, Cat. No. A610341-0100, Sangon). The beads were suspended in a 635 µl of 0.1 M MES, 125 µl of 0.2 M MES, and 3.08 mg 1-Ethyl-3-(3-dimethyl aminopropyl) carbodiimide hydrochloride (EDC, Cat. No. C600433-0025, Sangon; 3.08 mg). Then 7.5 µl beads were then divided into each well of a 96-well plate. Amino-modified oligonucleotides (2.5 µl, 50 µM in 0.1 M MES, all the sequences used are listed in Supplementary Dataset 9 and were synthesized by Sangon Biotech Co., Ltd. with high-performance liquid chromatography purification) were then added to each well. Incubating it for 20 min at room temperature and a 0.5 µl mix (6 mg EDC in 100 µl of 0.1 M MES) was distributed into each well. Then repeated once. The beads were collected and washed in 1 ml of 0.1 M phosphate-buffered saline (PBS, Cat. No. DT20012, Dianrui) containing 0.02% Tween 20 after 80 min vortexing and incubation. The supernatant was carefully removed using a magnetic grate. Finally, the beads were then washed twice in 1 ml TE (pH 8.0).

In the second split-pool, the beads were divided into each well of a new 96-well plate after washing with water. A 15 µl polymerase chain reaction (PCR) mix (1× Phanta Master Mix, Cat. No. P510-01, Vazyme) and 5 µM oligonucleotides were added to each well. The oligonucleotides in each well included a sequence with reverse complementarity to linker 1, a unique barcode and a linker 2 sequence, and the PCR program was as follows: 94 °C for 5 min; five cycles of 94 °C for 15 s, 48.8 °C for 4 min, and 72 °C for 4 min; and a 4 °C hold. Notably, the beads were mixed between denaturation (95 °C) and primer annealing (48.8 °C) in each cycle. After PCR program, the beads were collected in a 95 °C water bath for 6 min and separated using a magnet, removing complementary chains, two times.

In the third split-pool, the PCR program was as follows: 94 °C for 5 min, 48.8 °C for 20 min, and 72 °C for 4 min and a 4 °C hold. The oligonucleotides used in each well included a linker 2 reverse-complementary sequence, a unique barcode, a UMI sequence, and a poly-T tail. After the PCR program, the beads were collected and suspended in 200 µl exonuclease I mix (containing 1× exonuclease I buffer and 1 U/µl exonuclease I) and incubated at 37 °C for 15 min with a rotary mixer. Then the beads were washed with 200 µl TE-TW [10 mM Tris (pH 8.0) (Cat. No. 15568-025, Invitrogen), 1 mM EDTA (Cat. No. AM9260G, Invitrogen), 0.01% Tween 20 (Cat. No. A600560-0500, Sangon)], 200 µl of 10 mM Tris-HCl (pH 8.0), and 1 ml double-distilled water. Finally, the beads could be stored in 1 ml TE-TW for one month at 4 °C.

### Cell preparation

Adult *Xenopus laevis* tissues were minced into pieces (~1 mm) at room temperature using sterilized scissors. The tissue pieces were then transferred to a 15 ml centrifuge tube, washed with DPBS, and suspended in a 5 ml solution containing various dissociation enzymes for different amounts of time until no tissue fragments were visible. Details for single-cell isolation from different tissues are listed in Supplementary Dataset 1. To collect dissociated cells, the centrifuge tube was at 300 × *g* for 5 min at 4 °C, removed the supernatant, and resuspended in 2 ml DPBS. Then the dissociated cells were passed through a 70 µm strainer (Cat. No. F613462-9001, Sangon). After being washed twice (centrifuged at 300 × *g* for 5 min at 4 °C), the cells were resuspended at a density of 1 × 10^5^ cells/ml in DPBS.

Tadpoles whole body were also minced into pieces (~1 mm) at room temperature using scissors. The tadpole pieces were transferred to a 15 ml centrifuge tube and dissociated in 5 ml 1x TrypLE containing collagenase I (0.25 mg/ml), collagenase II (0.25 mg/ml), collagenase IV (0.25 mg/ml) and collagenase V (0.25 mg/ml). The remaining steps were consistent with adult *Xenopus laevis* tissues mentioned above.

### Cell collection and lysis

To eliminate the rate of the double cell during Microwell-seq, the cell concentration should be carefully controlled. The suitable cell concentration is about 100,000/ml (with 10% of the wells occupied by individual cells) and can be estimated using a hemocytometer. The cell suspension was pipetted onto the microwell plate and the plate was detected under a microscope to wash extra cells away. Then the bead suspension was loaded into the microwell plate which was placed on a magnet. The four sides of the microwell plate were removed and excess beads were washed away slightly. Cold lysis buffer [0.1 M Tris-HCl (pH 7.5) (Cat. No. 15567-027, Invitrogen), 0.5 M LiCl (Cat. No. A100416-0025, Sangon), 1% sodium dodecyl sulfate (SDS, Cat. No. B548118-0100, Sangon), 10 mM EDTA, and 5 mM dithiothreitol (Cat. No. A620058-0005, Sangon)] was pipetted over the surface of the microwell plate and incubated for 12 min. Next, the microwell plate was turned over and the beads were collected in a 1.5 ml RNase-free tube. The collected beads were washed once with 1 ml of 6× SSC (Cat. No. AM9770, Invitrogen), followed by 500 µl of 6× SSC and 200 µl of 50 mM Tris-HCl pH 8.0.

### Reverse transcription

Briefly, 20 µl RT mix [200 U SuperScript II reverse transcriptase (Cat. No. 2690 A, Takara), 1× Superscript II first-strand buffer (Cat. No. 2690 A, Takara), 20 U Murine RNase inhibitor (Cat. No. R301-2, Vazyme), 1 M betaine (Cat. No. 107-43-7, Sigma), 6 mM MgCl_2_ (Cat. No. AM9530G, Invitrogen), 2.5 mM dithiothreitol (Cat. No. A620058-0005, Sangon), 1 mM deoxynucleoside triphosphate (dNTP, Cat. No. P031-1, Vazyme), and 1 µM TSO primer] was added to the collected beads and were incubated at 42 °C for 90 min on a rotary mixer to keep even reaction. The beads were then washed with 200 µl TE-SDS (1× TE + 0.5% SDS) to inactivate reverse transcriptase.

### Exonuclease I treatment

The beads were washed with 200 µl TE-TW and 200 µl of 10 mM Tris-HCl (pH 8.0), pooled, and resuspended in 100 µl exonuclease I mix [1× exonuclease I buffer (Cat. No. B0293S, NEB), and 1 U/µl exonuclease I (Cat. No. M0293L, NEB)] and incubated at 37 °C for 30 min with mixing on a rotary mixer to remove single-stranded oligonucleotides without capturing mRNA. The beads were then washed once with TE-SDS, followed by 1 ml TE-TW and 200 µl of 10 mM Tris-HCl (pH 8.0).

### cDNA amplification

The beads were divided equally into four PCR tubes with 12.5 µl PCR mix [1× HiFi HotStart Readymix (Cat. No. KK2601, Kapa Biosystems.) and 0.1 µM TSO-PCR primer]. The PCR program was as follows: 98 °C for 3 min; six cycles of 98 °C for 20 s, 65 °C for 45 s, and 72 °C for 6 min; 72 °C for 10 min; and a 4 °C hold. The beads were mixed between denaturation (95 °C) and primer annealing (65 °C) in each cycle. Next, all PCR products were pooled together and VAHTS DNA Clean Beads (Cat. No. N411-03, Vazyme) were used to purify the pooling cDNA samples. To purify the DNA sample, a 25 µl PCR mix (1× HiFi HotStart Readymix and 0.1 µM TSO-PCR primer) was added again. The PCR program was as follows: 10 to 12 cycles of 98 °C for 3 min, 98 °C for 20 s, 67 °C for 20 s, and 72 °C for 6 min; 72 °C for 10 min; and a 4 °C hold. VAHTS DNA Clean beads were then used to purify the DNA sample and get the cDNA library.

### Transposase fragmentation and selective PCR

The purified cDNA libraries were fragmented using a customized transposase that carries two identical insertion sequences. The customized transposase was included in the TruePrep Homo-N7 DNA Library Prep Kit for Illumina (Cat. No. TD513, Vazyme) or TruePrep Homo-N7 DNA Library Prep Kit for MGI (Cat. No. L-N7E461L0, Vazyme). The fragmentation reaction was performed according to the instructions provided by the manufacturer. We used customed P5 primer (listed in Supplementary Dataset 9) and VAHTS RNA Adapters for Illumina (N8XX, Cat. No. TD203-1, Vazyme) or our MGI P7 primers (M8XX, listed in Supplementary Dataset 9) to specifically amplify fragments that contain the 3′ ends of transcripts. Other fragments will form self-loops, impeding their binding to PCR primers. The PCR program was as follows: 72 °C for 3 min; 98 °C for 1 min; five cycles of 98 °C for 15 s, 60 °C for 30 s, and 72 °C for 3 min; 72 °C for 5 min; and a 4 °C hold. The PCR product was purified using 0.9× VAHTS DNA Clean beads. Then, a 25 µL PCR mix (1×HiFi HotStart Readymix and 0.2 µM 2100 primer) was added to each sample. The PCR program was as follows: 95 °C for 3 min; five cycles of 98 °C for 20 s, 60 °C for 15 s, and 72 °C for 15 s; 72 °C for 3 min; and a 4 °C hold. To eliminate primer dimers and large fragments, 0.55-0.15× VAHTS DNA Clean beads were then used to purify the cDNA library. The size distribution of the products was analyzed on an Agilent 2100 bioanalyzer, and a peak in the 400 to 700 bp range was observed. Finally, the samples were subjected to sequencing on the Illumina HiSeq or MGI DNBSEQ-T7. For MGI sequencing, we applied the protocol provided by VAHTS Circularization Kit for MGI (Cat. No. NM201-01, Vazyme) to obtain single-stranded circular cDNA available for DNB (DNA Nanoball) generation. We also replaced the official R1 sequencing primers with our custom R1 sequencing primers A&B (listed in Supplementary Dataset 9) to ensure the completion of the sequencing.

### Quantification and statistical analysis

#### Preprocessing of Microwell-seq sequence data

Microwell-seq datasets were processed using the protocols described by ref. ^71^. The raw fastq-format sequencing data from a DNBSEQ-T7 were first split into i7 indexed sub-libraries using “splitBarcode [https://github.com/MGI-tech-bioinformatics/splitBarcode]”. Reads from *Xenopus laevis* were aligned to the *Xenopus laevis* v9.1 genome, using STAR^72^. The DGE data matrices were obtained using the modified Drop­seq tools (https://github.com/ggjlab/mca_data_analysis/tree/master/preprocessing/Drop-seq_tools-1.12/) and the corresponding protocol is available at http://mccarrolllab.org/dropseq/. The DGE data containing the top 30,000 cells sorted by the total number of transcripts were obtained after preprocessing. For quality control, we filtered out cells in which fewer than 500 transcripts were detected.

#### Correction of RNA contamination

During scRNA-seq data analysis, we commonly assume that all RNAs are endogenous to each individual cell. It has been recognized that some contaminating nonendogenous RNAs are also present even within datasets of the highest quality^73^. While applying Microwell-seq technology, there is a probability that a certain amount of cell-free RNA from the input solution admixed with the cell in a well are also sequenced. Although the amount of cell-free RNAs is very low compared with the total RNA content, it is still important to recognize and correct this contamination. To solve this problem, we strictly removed the batch gene background. We assumed that, for each batch of experiments, cell barcodes with fewer than 500 UMIs correspond to empty beads exposed to free RNA during cell lysis, RNA capture, and washing steps. Genes with extensive-expression in all beads were considered batch genes. The batch gene background value was defined as the average gene detection level for all cell barcodes with fewer than 500 UMIs, multiplied by the median of the fold difference between the detected gene expression of a cell and the average detected gene expression for beads with fewer than 500 UMIs and then rounded to the nearest integer. We subtracted the batch gene background for each cell from the digital expression matrix before performing the cross-tissue or cross-stage comparisons. We used the background-removed matrix to perform downstream analyses.

#### Filter out potential doublets

scRNA-seq data are commonly influenced by technical artifacts known as doublets, in which two or more different cells receive the same barcode, resulting in an aggregated transcriptome. We used the R package DoubletFinder^74^ to detect the potential doublets in each individual library. The overall doublet rate for our experiment was no more than 1.2%^12^. Approximately 5% of cells were labeled as doublets and were removed.

#### Cell clustering for single-cell datasets on a per-dataset basis

Seurat^75^ was used as a tool for cell clustering on a per-dataset basis. The data were log_2_ (counts per million (CPM)/100 + 1) transformed, and the number of UMIs and the percentage of mitochondrial gene content were regressed. A total of 2000 genes were selected through the “FindVariableFeatures” function by using the “vst” method and used as inputs for initial principal component analysis (PCA) and the number of principal components (PCs) used for nonlinear dimensional reduction (*t*-SNE). For clustering, we set different resolution parameters between 0.5 and 2.5 in the “FindCluster” function and chose the final cluster number by distinguishing differential genes among clusters. These parameters, including the resolution and number of PCs, were adjusted on a per-dataset basis.

#### Batch effect evaluation and clustering analysis for single-cell datasets on all dataset bases

The gene expression matrices for all single cells were merged together and fed to Seurat for clustering analysis. Briefly, the data were first processed by the “SCTransform” function. After that, processed data from larva were subjected to the Seurat standard procedure as described above. We chose 50 PCs for PCA and computed the neighborhood graph of cells. We then used the “FindCluster” function to cluster cells with a resolution of 0.8. Processed data from adults were fed to Scanpy^76^. We chose 100 PCs for PCA and computed the neighborhood graph of cells. We then used Leiden clustering to cluster cells with a resolution of 1 and neighbor number of 15. Finally, 57 clusters for larvae and 106 clusters for adults were produced, and marker genes were defined by the Wilcoxon rank-sum test. *t*-SNE was applied to visualize the single-cell transcriptional profile in 2D space. The Wilcoxon rank-sum test was used by running the ‘FindAllMarkers’ function in Seurat and the ‘rank genes groups’ function in Scanpy to find DEGs in each cluster. We annotated each cell type with marker genes selected based on an extensive literature review.

#### Collection of orthologous genes

Human, mouse, and zebrafish orthologous pairs were obtained from Ensembl v96 by BioMarkt. *Xenopus laevis* and human orthologous pairs were obtained from “Xenbase Home [http://www.xenbase.org/common/]”. In this study, we considered one-to-one orthologous and one-to-many paralogous pairs for further analysis.

#### Cross-species transcriptome comparison

Before transcriptome comparison, we collected human, mouse, and zebrafish cell-gene matrices and cell annotations from the previously published HCL, MCA, and ZCL datasets (https://db.cngb.org/HCL/; http://bis.zju.edu.cn/MCA; http://bis.zju.edu.cn/ZCA). We extracted the cells in the adult stage and divided the 102 human, 104 mouse, and 135 zebrafish cell clusters into 12 major cell lineages. To reduce the effects of noise and outliers, we randomly sampled 100,000 cells for each species, and we calculated the pseudocells, which was an average of every 50 cells from the same cell type, for further analysis. The gene expression data for each species underwent normalization to the sum of transcripts and multiplication by 100,000. The combined projection was visualized with the “SAMAP.scatter” function. To compare transcriptomes across species, we applied SAMap analysis between *Xenopus laevis* and humans, mice, and zebrafish. The cell type pairs with mapping scores higher than 0.1 were plotted between species by the package Circlize.

#### Lineage-specific transcription factor (TF) analysis

In this study, *Xenopus laevis* TFs were defined as the one-to-one orthologs of TFs found in *Xenopus tropicalis* downloaded from AnimalTFDB. We applied the same method used in previous research to judge cell-type-specific TFs for *Xenopus laevis*. We calculated the Jensen–Shannon divergence (JSD) for every TF expression status and defined the TF specificity score as 1 − $\sqrt{{JSD}}$. The *Z*-score-normalized TF specificity score was used to infer the specific TFs in each lineage. With reference to lineage-specific TF data for humans, mice, and zebrafish, we identified TFs conserved in four species for each lineage.

#### Identification of TFs that drive cell type mapping during metamorphosis

We define ${g}_{1}$ and ${g}_{2}$ to contain TFs from stage 1 and stage 2, respectively. Let ${X}_{{a}_{1}{a}_{2}}$ denote the set of all cells with cross-stage edges between cell types ${a}_{1}$ and ${a}_{2}$; JSD was used to evaluate cell-type-specific TFs for each stage such as stage1: ${Y}_{1,{g}_{1}}=1-\sqrt{{JSD}}$; We assign each TF a score: *h*_*g*_ = *T* (*S*(*Y*_1,*g*1_))∘ *T* (*S*(*Y*_2,*g*2_)), where *S*(*Z*) standardizes vector *Z* to have zero mean and unit variance, and *T*(*Z*) sets negative values in vector *Z* to zero in order to ignore weakly expressed genes. To be inclusive, the top 5 TFs according to ${h}_{g}$ are identified as “drive TFs”.

#### Gene expression trajectory analysis during metamorphosis

For module analysis, we used a similar method described in Kazer et al.^77^. We identified the DEGs between each stage using the “FindMarkers” function in Seurat. The parameters were logfc.threshold = 0.1, min.pct = 0.1 and min.diff.pct = -Inf. Then, we computed averaged expression values for each gene using the “AverageExpression” function in Seurat and grouped them into 15–30 clusters using the Mfuzz package in R with the fuzzy c-means algorithm^78^.

To determine whether cluster expression varied over time, every cell was scored for the genes within the cluster using the “AddModuleScore” function in Seurat. Then, 1000 two-sided Wilcoxon rank-sum tests were performed between the distribution of scores from 150–1000 cells at the first stage and the same number of cells from each other stage. For each stage, the *P* values from the 1000 tests were averaged. After FDR correction, if *q* < 1e-10 for any stage, the module was considered to vary significantly in expression in that stage.

Finally, these clusters were grouped into four modules using one-sided Wilcoxon rank-sum tests: module 1 was defined as the genes with the highest expression at NF59, module 2 was defined as the genes with the lowest expression at NF59, module 3 was defined as the genes that were consistently downregulated during metamorphosis, and module 4 was defined as the genes that were consistently upregulated.

#### Gene ontology analysis

Gene ontology analysis was performed on *Homo sapiens* orthologs using “g:profiler [https://biit.cs.ut.ee/gprofiler/gost]”, and all orthologous genes were taken as the universe. We selected the Benjamini–Hochberg FDR to compute multiple testing corrections and considered biological pathways with *p* values smaller than 0.05.

#### Statistics and reproducibility

No statistical method was used to predetermine sample size. No data were excluded from the analyses and all analyses were not randomized.

### Reporting summary

Further information on research design is available in the Nature Research Reporting Summary linked to this article.

## Supplementary information

Supplementary Information

Description of additional Supplementary File

Dataset 1

Dataset 2

Dataset 3

Dataset 4

Dataset 5

Dataset 6

Dataset 7

Dataset 8

Dataset 9

Reporting Summary

## Data Availability

The single-cell RNA-seq data generated in this study have been deposited in the NCBI Gene Expression Omnibus database under accession code “GSE195790”. The processed single-cell RNA-seq data were available at “Figshare [10.6084/m9.figshare.19152839]” and on the “XCL website [http://bis.zju.edu.cn/XCL/]”. All other relevant data supporting the key findings of this study are available within the article and its Supplementary Information files or from the corresponding author upon reasonable request.

## Code availability

The detail analyses code is available at “github [https://github.com/ggjlab/XCL]”^79^.

## References

[1] Briggs, J. A. et al. The dynamics of gene expression in vertebrate embryogenesis at single-cell resolution. *Science*10.1126/science.aar5780 (2018). 10.1126/science.aar5780 PMC6038144 29700227

[2] Bright AR (2021). Combinatorial transcription factor activities on open chromatin induce embryonic heterogeneity in vertebrates. EMBO J..

[3] Sonam, S., Bangru, S., Perry, K. J., Kalsotra, A. & Henry, J. J. Single-cell analyses of the corneal epithelium: Unique cell types 1 and gene expression profiles. Preprint at *bioRxiv*10.1101/2020.08.06.240036 (2020).

[4] Corkins, M. E. et al. A comparative study of cellular diversity between the Xenopus pronephric and mouse metanephric nephron. Preprint at *bioRxiv*10.1101/2022.01.11.475739 (2022). 10.1016/j.kint.2022.07.027 PMC9822858 36055600

[5] Lin T-Y (2021). Fibroblast dedifferentiation as a determinant of successful regeneration. Dev. Cell.

[6] Aztekin, C. et al. Secreted inhibitors drive the loss of regeneration competence in Xenopus limbs. *Development*10.1242/dev.199158 (2021). 10.1242/dev.199158 PMC8217717 34105722

[7] Kakebeen, A. D., Chitsazan, A. D., Williams, M. C., Saunders, L. M. & Wills, A. E. Chromatin accessibility dynamics and single cell RNA-Seq reveal new regulators of regeneration in neural progenitors. *Elife*10.7554/eLife.52648 (2020). 10.7554/eLife.52648 PMC7250574 32338593

[8] Aztekin C (2019). Identification of a regeneration-organizing cell in the Xenopus tail. Science.

[9] Pelzer, D. et al. Foxm1 regulates neural progenitor fate during spinal cord regeneration. *EMBO Rep.*10.15252/embr.202050932 (2021). 10.15252/embr.202050932 PMC8419688 34427977

[10] Han XP (2020). Construction of a human cell landscape at single-cell level. Nature.

[11] Qu, J. et al. A reference single-cell regulomic and transcriptomic map of cynomolgus monkeys. *Nat. Commun.* **13**, 10.1038/s41467-022-31770-x (2022). 10.1038/s41467-022-31770-x PMC9279386 35831300

[12] Han X (2018). Mapping the mouse cell atlas by microwell-seq. Cell.

[13] Spanjaard B (2018). Simultaneous lineage tracing and cell-type identification using CRISPR-Cas9-induced genetic scars. Nat. Biotechnol..

[14] Li H (2022). Fly cell atlas: a single-nucleus transcriptomic atlas of the adult fruit fly. Science.

[15] Cao C (2019). Comprehensive single-cell transcriptome lineages of a proto-vertebrate. Nature.

[16] Cao J (2017). Comprehensive single-cell transcriptional profiling of a multicellular organism. Science.

[17] Fincher, C. T., Wurtzel, O., de Hoog, T., Kravarik, K. M. & Reddien, P. W. Cell type transcriptome atlas for the planarian Schmidtea mediterranea. *Science*10.1126/science.aaq1736 (2018). 10.1126/science.aaq1736 PMC6563842 29674431

[18] Levy S (2021). A stony coral cell atlas illuminates the molecular and cellular basis of coral symbiosis, calcification, and immunity. Cell.

[19] Marshall LN (2019). Stage-dependent cardiac regeneration in Xenopus is regulated by thyroid hormone availability. Proc. Natl Acad. Sci. USA.

[20] Tata JR (1993). Gene expression during metamorphosis: an ideal model for post-embryonic development. Bioessays.

[21] Sin WC, Haas K, Ruthazer ES, Cline HT (2002). Dendrite growth increased by visual activity requires NMDA receptor and Rho GTPases. Nature.

[22] Satija R, Farrell JA, Gennert D, Schier AF, Regev A (2015). Spatial reconstruction of single-cell gene expression data. Nat. Biotechnol..

[23] Crow M, Paul A, Ballouz S, Huang ZJ, Gillis J (2018). Characterizing the replicability of cell types defined by single cell RNA-sequencing data using MetaNeighbor. Nat. Commun..

[24] Zhang C (2020). The transcription factor NKX2-2 regulates oligodendrocyte differentiation through domain-specific interactions with transcriptional corepressors. J. Biol. Chem..

[25] Kohler S, Winkler U, Hirrlinger J (2021). Heterogeneity of astrocytes in grey and white matter. Neurochem. Res..

[26] Zuasti A, Jimenez-Cervantes C, Garcia-Borron JC, Ferrer C (1998). The melanogenic system of Xenopus laevis. Arch. Histol. Cytol..

[27] Tarashansky, A. J. et al. Mapping single-cell atlases throughout Metazoa unravels cell type evolution. *Elife*10.7554/eLife.66747 (2021). 10.7554/eLife.66747 PMC8139856 33944782

[28] Wang J (2021). Tracing cell-type evolution by cross-species comparison of cell atlases. Cell Rep..

[29] Jiang, M. et al. Characterization of the zebrafish cell landscape at single-cell resolution. *Front. Cell Dev. Biol.*10.3389/fcell.2021.743421 (2021). 10.3389/fcell.2021.743421 PMC8517238 34660600

[30] Hadji-Azimi I, Coosemans V, Canicatti C (1987). Atlas of adult Xenopus laevis laevis hematology. Dev. Comp. Immunol..

[31] Nogawa-Kosaka N (2011). Identification of erythroid progenitors induced by erythropoietic activity in Xenopus laevis. J. Exp. Biol..

[32] Popescu DM (2019). Decoding human fetal liver haematopoiesis. Nature.

[33] Geirsdottir L (2019). Cross-species single-cell analysis reveals divergence of the primate microglia program. Cell.

[34] Sueda R, Kageyama R (2020). Regulation of active and quiescent somatic stem cells by Notch signaling. Dev. Growth Differ..

[35] Stoeck A (2014). Discovery of biomarkers predictive of GSI response in triple-negative breast cancer and adenoid cystic carcinoma. Cancer Disco..

[36] Phinney DG (2011). Twist, epithelial-to-mesenchymal transition, and stem cells. Stem Cells.

[37] Coppiello G (2015). Meox2/Tcf15 heterodimers program the heart capillary endothelium for cardiac fatty acid uptake. Circulation.

[38] Gohn CR, Blue EK, Sheehan BM, Varberg KM, Haneline LS (2017). Mesenchyme homeobox 2 enhances migration of endothelial colony forming cells exposed to intrauterine diabetes mellitus. J. Cell Physiol..

[39] Doty RT (2019). Single-cell analyses demonstrate that a heme-GATA1 feedback loop regulates red cell differentiation. Blood.

[40] Horie K (2019). Down-regulation of GATA1-dependent erythrocyte-related genes in the spleens of mice exposed to a space travel. Sci. Rep..

[41] Daly ME (2017). Transcription factor defects causing platelet disorders. Blood Rev..

[42] Milner JJ (2017). Runx3 programs CD8(+) T cell residency in non-lymphoid tissues and tumours. Nature.

[43] Sekizar S (2015). Remyelination by resident oligodendrocyte precursor cells in a Xenopus laevis inducible model of demyelination. Dev. Neurosci..

[44] Nitta KR (2006). Expression of Sox1 during Xenopus early embryogenesis. Biochem. Biophys. Res. Commun..

[45] Baltzinger M, Mager-Heckel AM, Remy P (1999). Xl erg: expression pattern and overexpression during development plead for a role in endothelial cell differentiation. Dev. Dyn..

[46] Cermenati S (2008). Sox18 and Sox7 play redundant roles in vascular development. Blood.

[47] Yang WY (2015). Coronary risk in relation to genetic variation in MEOX2 and TCF15 in a Flemish population. BMC Genet..

[48] Betancur P, Bronner-Fraser M, Sauka-Spengler T (2010). Genomic code for Sox10 activation reveals a key regulatory enhancer for cranial neural crest. Proc. Natl Acad. Sci. USA.

[49] Zhang L (2015). Small molecules efficiently reprogram human astroglial cells into functional neurons. Cell Stem Cell.

[50] Jakovcevski I, Zecevic N (2005). Olig transcription factors are expressed in oligodendrocyte and neuronal cells in human fetal CNS. J. Neurosci..

[51] Papes F (2022). Transcription factor 4 loss-of-function is associated with deficits in progenitor proliferation and cortical neuron content. Nat. Commun..

[52] Yuan F (2020). LHX6 is essential for the migration of human pluripotent stem cell-derived GABAergic interneurons. Protein Cell.

[53] Dai JX, Bercury KK, Ahrendsen JT, Macklin WB (2015). Olig1 function is required for oligodendrocyte differentiation in the mouse brain. J. Neurosci..

[54] Bronchain OJ (2007). The olig family: phylogenetic analysis and early gene expression in Xenopus tropicalis. Dev. Genes Evol..

[55] Furlow JD, Neff ES (2006). A developmental switch induced by thyroid hormone: Xenopus laevis metamorphosis. Trends Endocrinol. Metab..

[56] Heimeier RA, Das B, Buchholz DR, Fiorentino M, Shi YB (2010). Studies on Xenopus laevis intestine reveal biological pathways underlying vertebrate gut adaptation from embryo to adult. Genome Biol..

[57] Brown DD, Cai L (2007). Amphibian metamorphosis. Dev. Biol..

[58] Schreiber AM, Cai L, Brown DD (2005). Remodeling of the intestine during metamorphosis of Xenopus laevis. Proc. Natl Acad. Sci. USA.

[59] Ju JY (2014). Human fetal globin gene expression is regulated by LYAR. Nucleic Acids Res..

[60] Kassouf MT (2010). Genome-wide identification of TAL1’s functional targets: Insights into its mechanisms of action in primary erythroid cells. Genome Res..

[61] Mukhi S, Cai LQ, Brown DD (2010). Gene switching at Xenopus laevis metamorphosis. Dev. Biol..

[62] Maniatis GM, Ingram VM (1971). Erythropoiesis during amphibian metamorphosis: I. Site of maturation of erythrocytes in Rana catesbeiana. J. Cell Biol..

[63] Jurd RD, Maclean N (1970). An immunofluorescent study of the haemoglobins in metamorphosing Xenopus laevis. J. Embryol. Exp. Morphol..

[64] Widmer HJ, Hosbach HA, Weber R (1983). Globin gene expression in Xenopus laevis: anemia induces precocious globin transition and appearance of adult erythroblasts during metamorphosis. Dev. Biol..

[65] Flajnik MF (1986). Major histocompatibility complex-encoded class I molecules are absent in immunologically competent Xenopus before metamorphosis. J. Immunol..

[66] Wong ML, Dong C, Maestre-Mesa J, Licinio J (2008). Polymorphisms in inflammation-related genes are associated with susceptibility to major depression and antidepressant response. Mol. Psychiatr..

[67] Flajnik MF, Dupasquier L (1988). Mhc class-I antigens as surface-markers of adult erythrocytes during the metamorphosis of xenopus. Dev. Biol..

[68] Flores IL (2016). EEF1D modulates proliferation and epithelial-mesenchymal transition in oral squamous cell carcinoma. Clin. Sci..

[69] Liu Y (2020). LAIR-1 suppresses cell growth of ovarian cancer cell via the PI3K-AKT-mTOR pathway. Aging.

[70] Perry SF, Sander M (2004). Reconstructing the evolution of the respiratory apparatus in tetrapods. Respir. Physiol. Neurobiol..

[71] Han X (2018). Mapping the mouse cell atlas by microwell-seq. Cell.

[72] Dobin A (2013). STAR: ultrafast universal RNA-seq aligner. Bioinformatics.

[73] Zheng GX (2017). Massively parallel digital transcriptional profiling of single cells. Nat. Commun..

[74] McGinnis CS, Murrow LM, Gartner ZJ (2019). DoubletFinder: doublet detection in single-cell RNA sequencing data using artificial nearest neighbors. Cell Syst..

[75] Stuart T (2019). Comprehensive integration of single-cell data. Cell.

[76] Wolf FA, Angerer P, Theis FJ (2018). SCANPY: large-scale single-cell gene expression data analysis. Genome Biol..

[77] Kazer SW (2020). Integrated single-cell analysis of multicellular immune dynamics during hyperacute HIV-1 infection. Nat. Med..

[78] Kumar L, E Futschik M (2007). Mfuzz: a software package for soft clustering of microarray data. Bioinformation.

[79] Liao, Y. et al. Cell landscape of larval and adult Xenopus laevis at single-cell resolution (2022). 10.1038/s41467-022-31949-2 PMC9314398 35879314

