# Publisher Correction: Cell landscape of larval and adult *Xenopus laevis* at single-cell resolution

**DOI:** 10.1038/s41467-022-32733-y

**Published:** 2022-09-01

**Authors:** Yuan Liao, Lifeng Ma, Qile Guo, Weigao E, Xing Fang, Lei Yang, Fanwei Ruan, Jingjing Wang, Peijing Zhang, Zhongyi Sun, Haide Chen, Zhongliang Lin, Xueyi Wang, Xinru Wang, Huiyu Sun, Xiunan Fang, Yincong Zhou, Ming Chen, Wanhua Shen, Guoji Guo, Xiaoping Han

**Affiliations:** 1 Center for Stem Cell and Regenerative Medicine, and Bone Marrow Transplantation Center of the First Affiliated Hospital, Zhejiang University School of Medicine, Hangzhou, 310000 China; 2 Zhejiang Provincial Key Lab for Tissue Engineering and Regenerative Medicine, Dr. Li Dak Sum & Yip Yio Chin Center for Stem Cell and Regenerative Medicine, Hangzhou, Zhejiang 310058 China; 3 ZJU-UOE Institute, Zhejiang University, School of Medicine, Haining, Zhejiang China; 4 Liangzhu Laboratory, Zhejiang University Medical Center, Hangzhou, Zhejiang 311121 China; 5 College of Life Sciences, Zhejiang University, Hangzhou, 310003 China; 6 Zhejiang Key Laboratory of Organ Development and Regeneration, College of Life and Environmental Sciences, Hangzhou Normal University, Hangzhou, Zhejiang China

**Keywords:** RNA sequencing, Developmental biology, Biological models, Gene expression analysis

Correction to: *Nature Communications* 10.1038/s41467-022-31949-2, published online 25 July 2022

The original version of this Article contained an error in the punctuation of the last name of the fourth author, which incorrectly read ‘<Weigao E.>.’ The correct version contains no punctuation and states ‘<Weigao E>’. This has been corrected in both the PDF and HTML versions of the Article.

