# Supplementary material for: Cell landscape of larval and adult Xenopus laevis at single-cell resolution
Source: Nat Commun. 2022 Jul 25;13:4306. doi: 10.1038/s41467-022-31949-2 (PMC9314398; doi:10.1038/s41467-022-31949-2)
Supplement: Supplementary file 1 — Supplementary Information [file 41467_2022_31949_MOESM1_ESM.pdf]

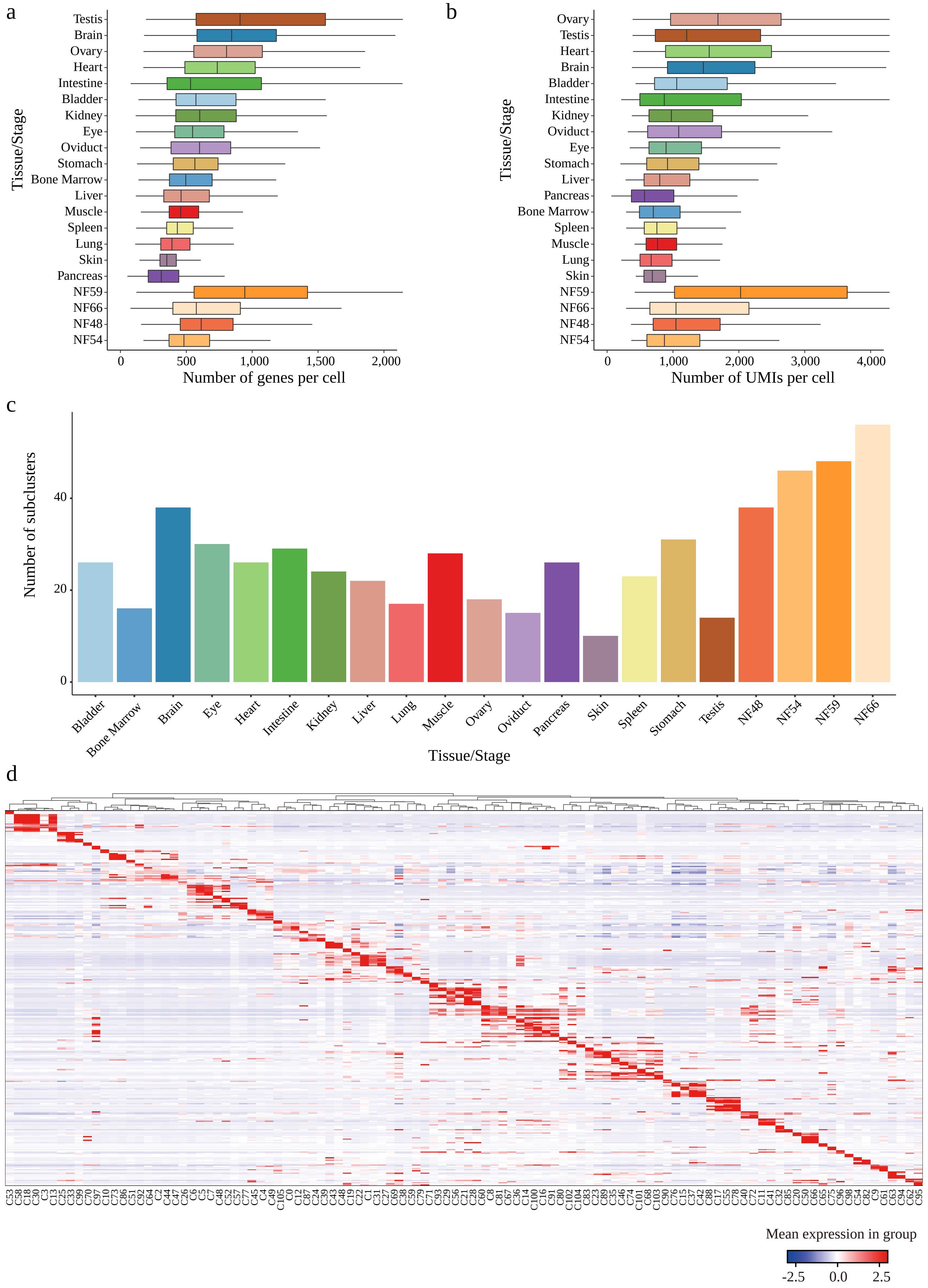
a
b
c
d
Tissue/Stage
Testis
Brain
Ovary
Heart
Intestine
Bladder
Kidney
Eye
Oviduct
Stomach
Bone Marrow
Liver
Muscle
Spleen
Lung
Skin
Pancreas
NF59
NF66
NF48
NF54
0
500
1,000
1,500
2,000
Number of genes per cell
1,000
2,000
3,000
4,000
Number of UMIs per cell
Number of subclusters
0
20
40
Mean expression in group
-2.5
0.0
2.5

Supplementary Figure 1. Details of XCL construction. a, Number of genes per cell detected for each tissue/stage in the XCL. b, Number of UMIs per cell detected for each tissue/stage in the XCL. c, Number of subclusters detected for each tissue/stage in the XCL. d, Heatmap showing representative gene expression in each cell cluster in the XCL.

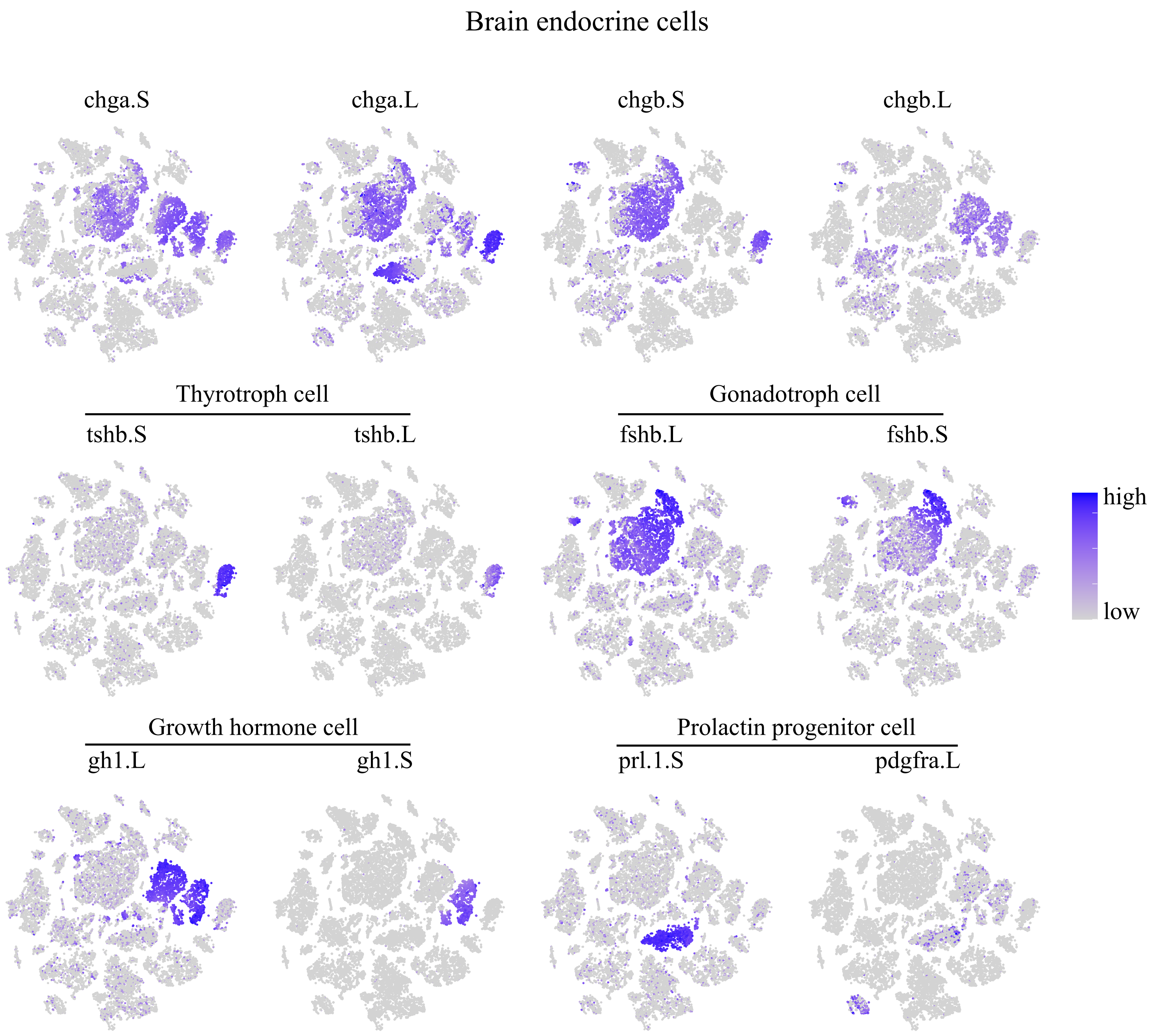
Brain endocrine cells
chga.S
chga.L
chgb.S
chgb.L
Thyrotroph cell
tshb.S
tshb.L
Gonadotroph cell
fshb.L
fshb.S
high
low
Growth hormone cell
gh1.L
gh1.S
Prolactin progenitor cell
prl.1.S
pdgfra.L

Supplementary Figure 2. Representative gene expression in endocrine cells of the *Xenopus* brain. Feature plot in the t-SNE map of adult *Xenopus* brain single-cell data. Kinds of endocrine cells are colored according to the expression of the indicated marker genes: co-expressed high levels of *chga* and *chgb* but different hormone-associated genes.

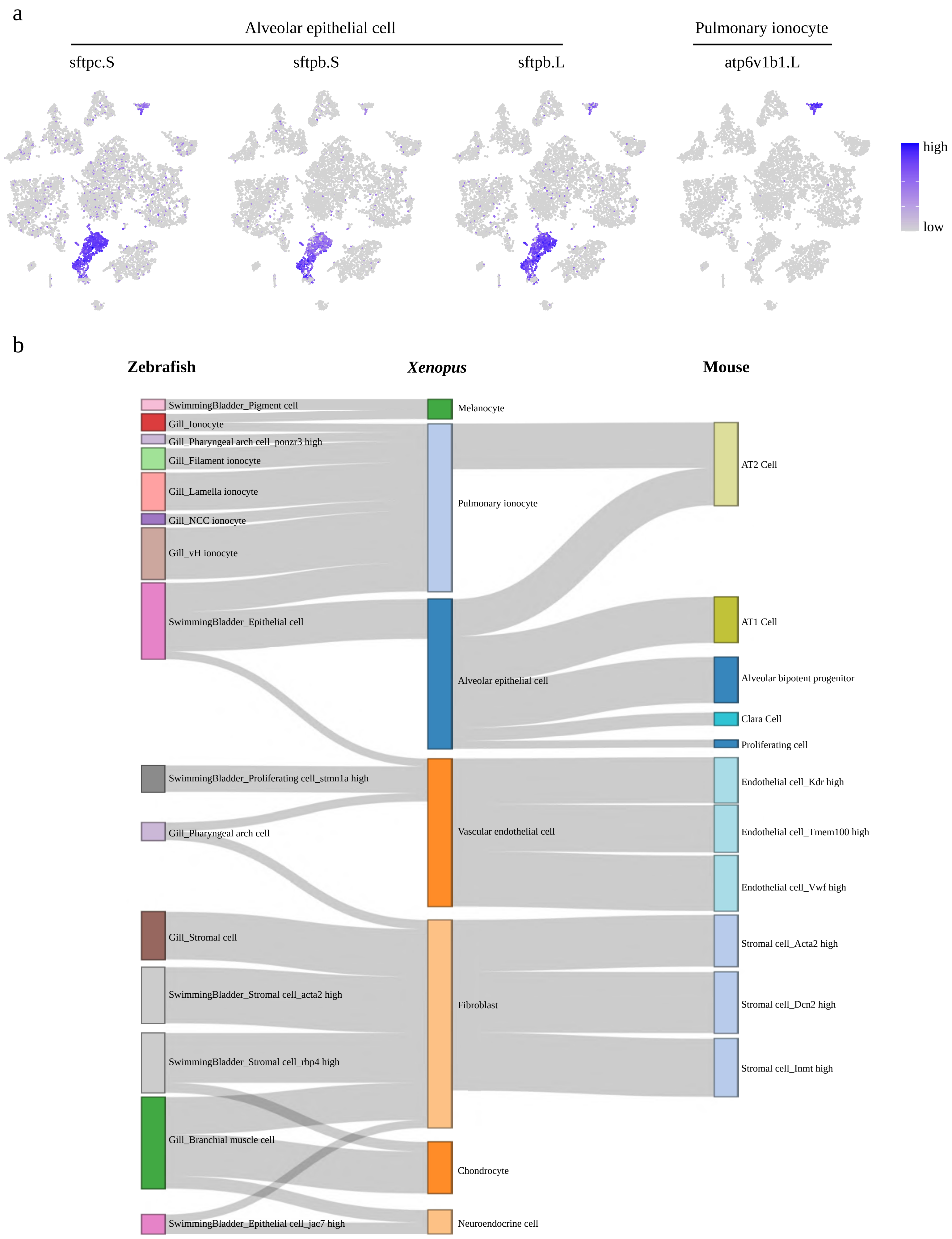
a
Alveolar epithelial cell
Pulmonary ionocyte
sftpc.S
sftpb.S
sftpb.L
atp6v1b1.L
high
low
b
Zebrafish
Xenopus
Mouse
SwimmingBladder_Pigment cell
Gill_Ionocyte
Gill_Pharyngeal arch cell_ponzr3 high
Gill_Filament ionocyte
Gill_Lamella ionocyte
Gill_NCC ionocyte
Gill_vH ionocyte
SwimmingBladder_Epithelial cell
SwimmingBladder_Proliferating cell_stmn1a high
Gill_Pharyngeal arch cell
Gill_Stromal cell
SwimmingBladder_Stromal cell_acta2 high
SwimmingBladder_Stromal cell_rbp4 high
Gill_Branchial muscle cell
SwimmingBladder_Epithelial cell_jac7 high
Melanocyte
Pulmonary ionocyte
Alveolar epithelial cell
Vascular endothelial cell
Fibroblast
Chondrocyte
Neuroendocrine cell
AT2 Cell
AT1 Cell
Alveolar bipotent progenitor
Clara Cell
Proliferating cell
Endothelial cell_Kdr high
Endothelial cell_Tmem100 high
Endothelial cell_Vwf high
Stromal cell_Acta2 high
Stromal cell_Dcn2 high
Stromal cell_Inmt high

Supplementary Figure 3. Details of cellular heterogeneity in adult *Xenopus* lung. a, Feature plot in the t-SNE map of adult *Xenopus* lung single-cell data. Cells are colored according to the expression of the indicated marker genes. b, Sankey plot showing the relationships among the *Xenopus* lung, mouse lung, zebrafish gill and swim bladder. Different cell clusters in each species are marked in different color boxes.

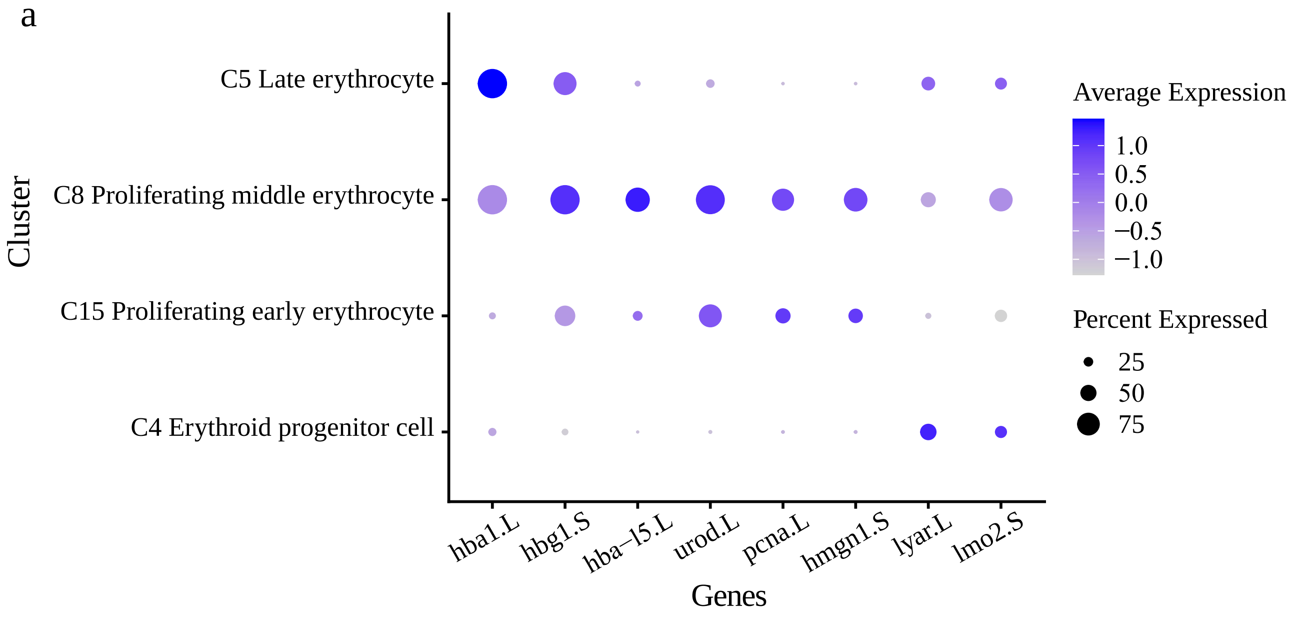

a
Cluster
C5 Late erythrocyte
C8 Proliferating middle erythrocyte
C15 Proliferating early erythrocyte
C4 Erythroid progenitor cell
hba1.L
hbg1.S
hba−15.L
urod.L
pcna.L
hmgn1.S
lyar.L
lmo2.S
Genes
Average Expression
1.0
0.5
0.0
−0.5
−1.0
Percent Expressed
25
50
75

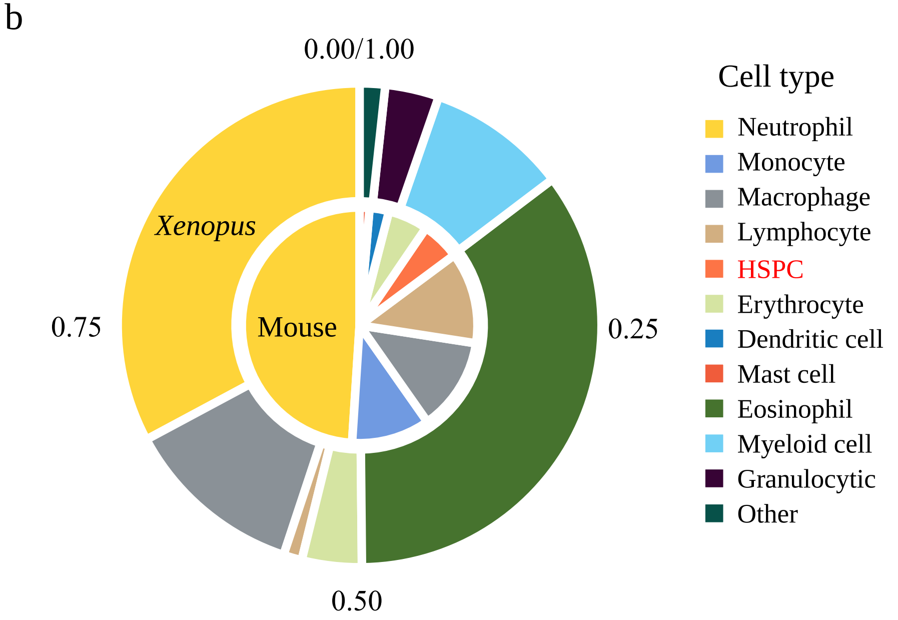

b
0.00/1.00
0.25
0.50
0.75
Xenopus
Mouse
Cell type
Neutrophil
Monocyte
Macrophage
Lymphocyte
HSPC
Erythrocyte
Dendritic cell
Mast cell
Eosinophil
Myeloid cell
Granulocytic
Other

Supplementary Figure 4. Details of cellular heterogeneity in adult *Xenopus* liver and bone marrow. a, Representative gene expression in each cluster of liver erythrocytes in different stages. The size of the dot encodes the percentage of cells within a cell type, and the color encodes the average expression level. b, Cell proportions of bone marrow in *Xenopus* (outer circle) and mouse (inside circle). Cell types are labeled in different colors.

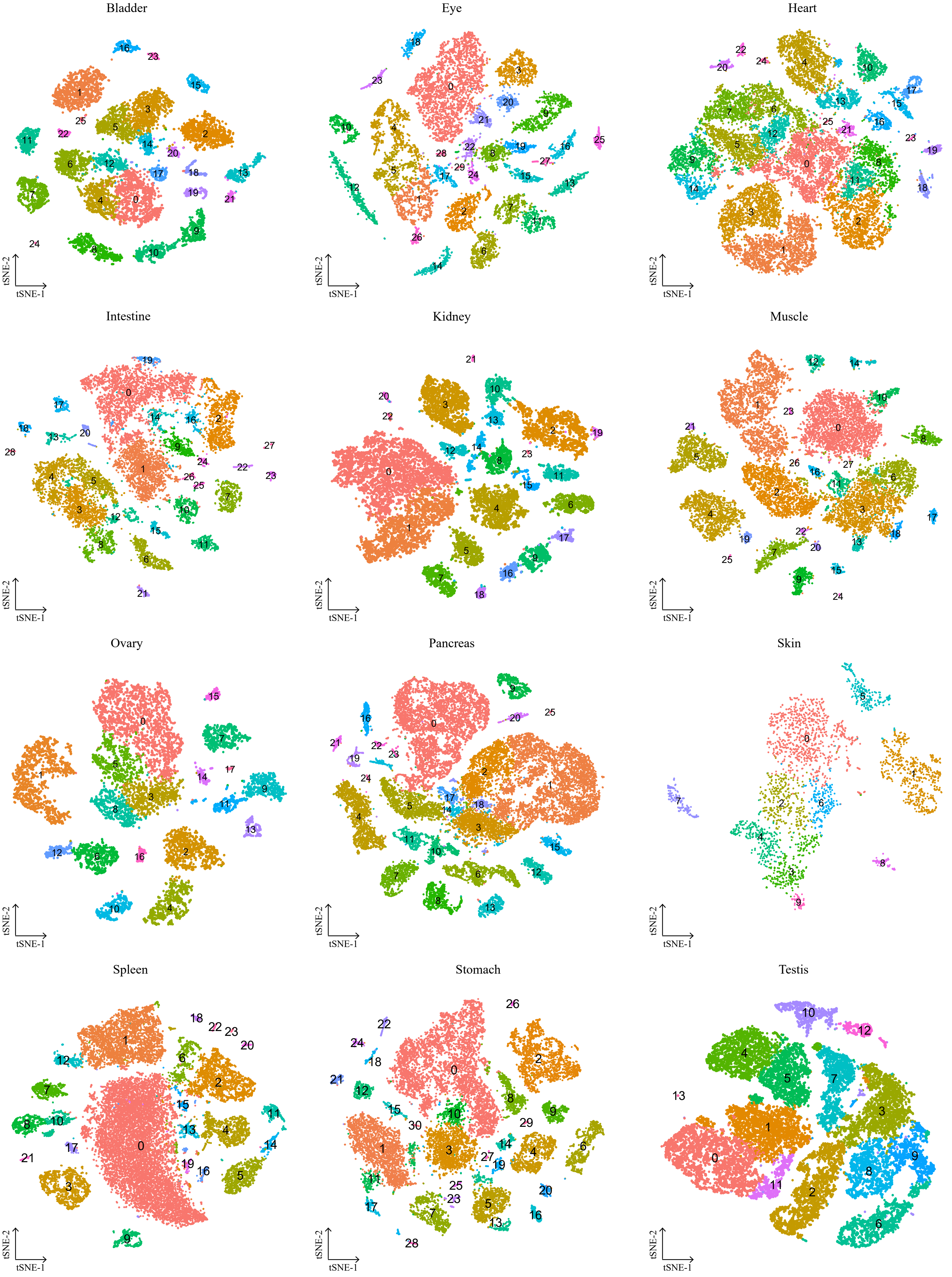
Bladder
Eye
Heart
Intestine
Kidney
Muscle
Ovary
Pancreas
Skin
Spleen
Stomach
Testis
tSNE-1
tSNE-2

Supplementary Figure 5. t-SNE analysis of XCL tissues collected from adult *Xenopus*. t-SNE maps of single-cell data from bladder, eye, heart, intestine, kidney, muscle, ovary, pancreas, skin, spleen, stomach and testis. Cells are colored by cell-type cluster.

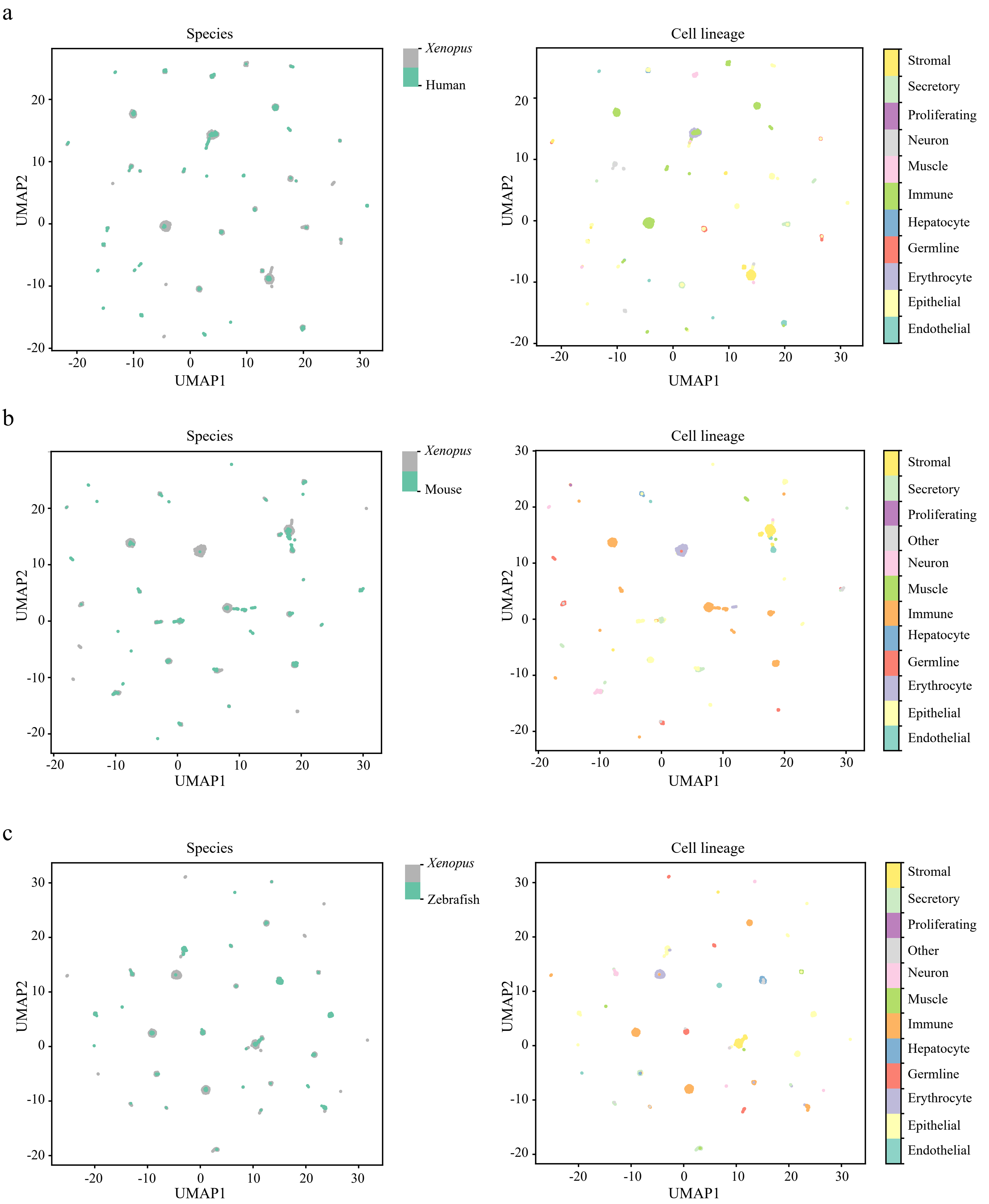
a
Species
Xenopus
Human
UMAP2
UMAP1
Cell lineage
Stromal
Secretory
Proliferating
Neuron
Muscle
Immune
Hepatocyte
Germline
Erythrocyte
Epithelial
Endothelial
b
Species
Xenopus
Mouse
UMAP2
UMAP1
Cell lineage
Stromal
Secretory
Proliferating
Other
Neuron
Muscle
Immune
Hepatocyte
Germline
Erythrocyte
Epithelial
Endothelial
c
Species
Xenopus
Zebrafish
UMAP2
UMAP1
Cell lineage
Stromal
Secretory
Proliferating
Other
Neuron
Muscle
Immune
Hepatocyte
Germline
Erythrocyte
Epithelial
Endothelial

Supplementary Figure 6. The UMAP projection of combined species. The UMAP projection of the combined human and *Xenopus* (a), mouse and *Xenopus* (b), zebrafish and *Xenopus* (c) manifolds are visualized, colored by species (left) and lineages (right) respectively. Cells are colored by cell-type cluster.

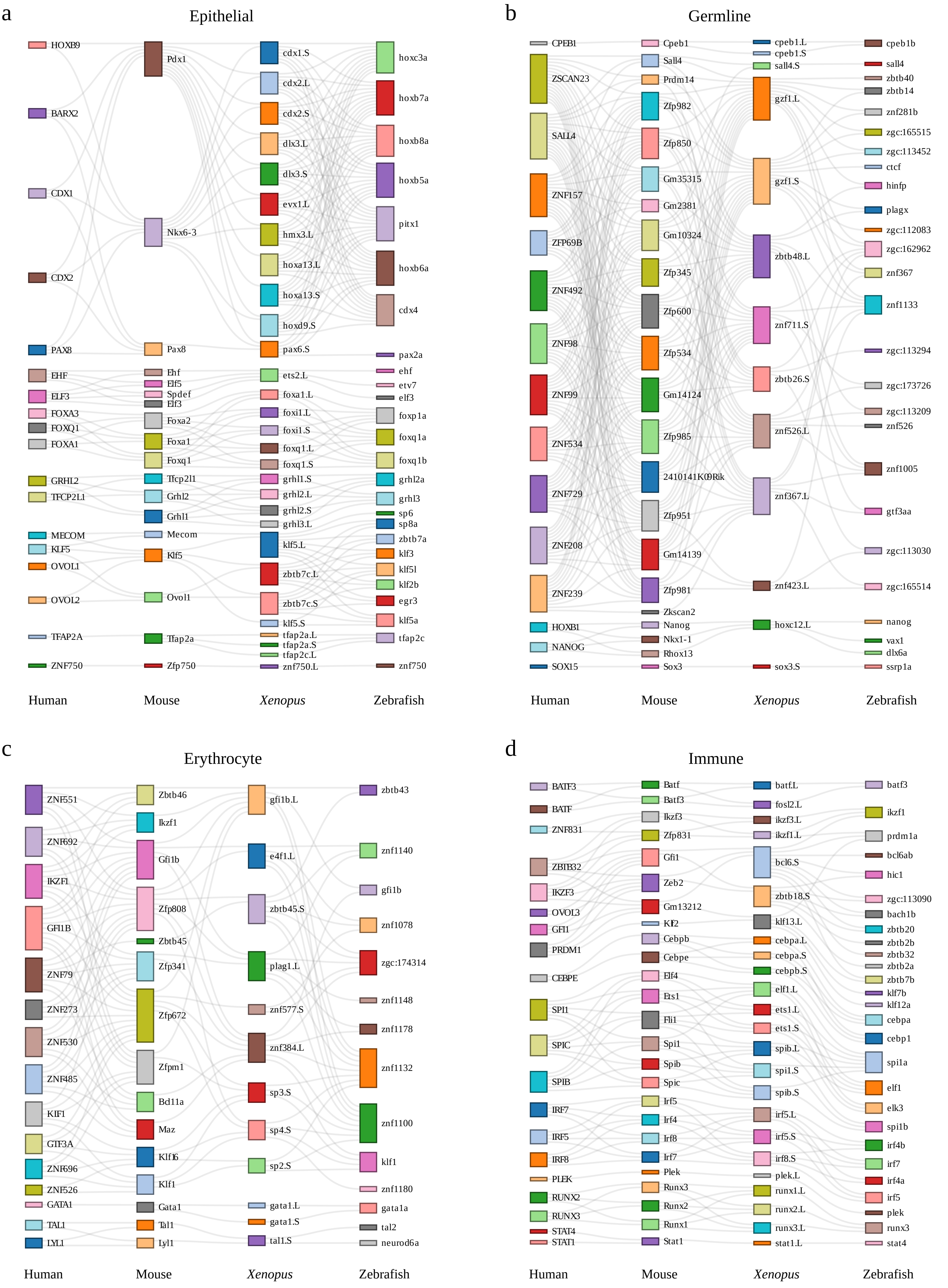
a
Epithelial
HOXB9
BARX2
CDX1
CDX2
PAX8
EHF
ELF3
FOXA3
FOXQ1
FOXA1
GRHL2
TFCP2L1
MECOM
KLF5
OVOL1
OVOL2
TFAP2A
ZNF750
Pdx1
Nkx6-3
Pax8
Ehf
Elf5
Spdef
Elf3
Foxa2
Foxa1
Foxq1
Tfcp2l1
Grhl2
Grhl1
Mecom
Klf5
Ovol1
Tfap2a
Zfp750
cdx1.S
cdx2.L
cdx2.S
dlx3.L
dlx3.S
evx1.L
hmx3.L
hoxa13.L
hoxa13.S
hoxd9.S
pax6.S
ets2.L
foxa1.L
foxi1.L
foxi1.S
foxq1.L
foxq1.S
grhl1.S
grhl2.L
grhl2.S
grhl3.L
klf5.L
zbtb7c.L
zbtb7c.S
klf5.S
tfap2a.L
tfap2a.S
tfap2c.L
znf750.L
hoxc3a
hoxb7a
hoxb8a
hoxb5a
pitx1
hoxb6a
cdx4
pax2a
ehf
etv7
elf3
foxp1a
foxq1a
foxq1b
grhl2a
grhl3
sp6
sp8a
zbtb7a
klf3
klf5l
klf2b
egr3
klf5a
tfap2c
znf750
Human
Mouse
Xenopus
Zebrafish
b
Germline
CPEB1
ZSCAN23
SALL4
ZNF157
ZFP69B
ZNF492
ZNF98
ZNF99
ZNF534
ZNF729
ZNF208
ZNF239
HOXB1
NANOG
SOX15
Cpeb1
Sall4
Prdm14
Zfp982
Zfp850
Gm35315
Gm2381
Gm10324
Zfp345
Zfp600
Zfp534
Gm14124
Zfp985
2410141K09Rik
Zfp951
Gm14139
Zfp981
Zkscan2
Nanog
Nkx1-1
Rhox13
Sox3
cpeb1.L
cpeb1.S
sall4.S
gzf1.L
gzf1.S
zbtb48.L
znf711.S
zbtb26.S
znf526.L
znf367.L
znf423.L
hoxc12.L
sox3.S
cpeb1b
sall4
zbtb40
zbtb14
znf281b
zgc:165515
zgc:113452
ctcf
hinfp
plagx
zgc:112083
zgc:162962
znf367
znf1133
zgc:113294
zgc:173726
zgc:113209
znf526
znf1005
gtf3aa
zgc:113030
zgc:165514
nanog
vax1
dlx6a
ssrp1a
Human
Mouse
Xenopus
Zebrafish
c
Erythrocyte
ZNF551
ZNF692
IKZF1
GFI1B
ZNF79
ZNF273
ZNF530
ZNF485
KLF1
GTF3A
ZNF696
ZNF526
GATA1
TAL1
LYL1
Zbtb46
Ikzf1
Gfi1b
Zfp808
Zbtb45
Zfp341
Zfp672
Zfpm1
Bcl11a
Maz
Klf16
Klf1
Gata1
Tal1
Lyl1
gfi1b.L
e4f1.L
zbtb45.S
plag1.L
znf577.S
znf384.L
sp3.S
sp4.S
sp2.S
gata1.L
gata1.S
tal1.S
zbtb43
znf1140
gfi1b
znf1078
zgc:174314
znf1148
znf1178
znf1132
znf1100
klf1
znf1180
gata1a
tal2
neurod6a
Human
Mouse
Xenopus
Zebrafish
d
Immune
BATF3
BATF
ZNF831
ZBTB32
IKZF3
OVOL3
GFI1
PRDM1
CEBPE
SPI1
SPIC
SPIB
IRF7
IRF5
IRF8
PLEK
RUNX2
RUNX3
STAT4
STAT1
Batf
Batf3
Ikzf3
Zfp831
Gfi1
Zeb2
Gm13212
Klf2
Cebpb
Cebpe
Elf4
Ets1
Fli1
Spi1
Spib
Spic
Irf5
Irf4
Irf8
Irf7
Plek
Runx3
Runx2
Runx1
Stat1
batf.L
fosl2.L
ikzf3.L
ikzf1.L
bcl6.S
zbtb18.S
klf13.L
cebpa.L
cebpa.S
cebpb.S
elf1.L
ets1.L
ets1.S
spib.L
spi1.S
spib.S
irf5.L
irf5.S
irf8.S
plek.L
runx1.L
runx2.L
runx3.L
stat1.L
batf3
ikzf1
prdm1a
bcl6ab
hic1
zgc:113090
bach1b
zbtb20
zbtb2b
zbtb32
zbtb2a
zbtb7b
klf7b
klf12a
cebpa
cebp1
spi1a
elf1
elk3
spi1b
irf4b
irf7
irf4a
irf5
plek
runx3
stat4
Human
Mouse
Xenopus
Zebrafish

Supplementary Figure S7. The common lineage-specific TFs for each lineage. Sankey plot showing common lineage-specific TFs in epithelial (a), germline (b), erythrocyte (c) and immune (d). Different TFs in each species are marked in different color boxes. Homologous TFs between species obtained from SAMap are connected by lines.

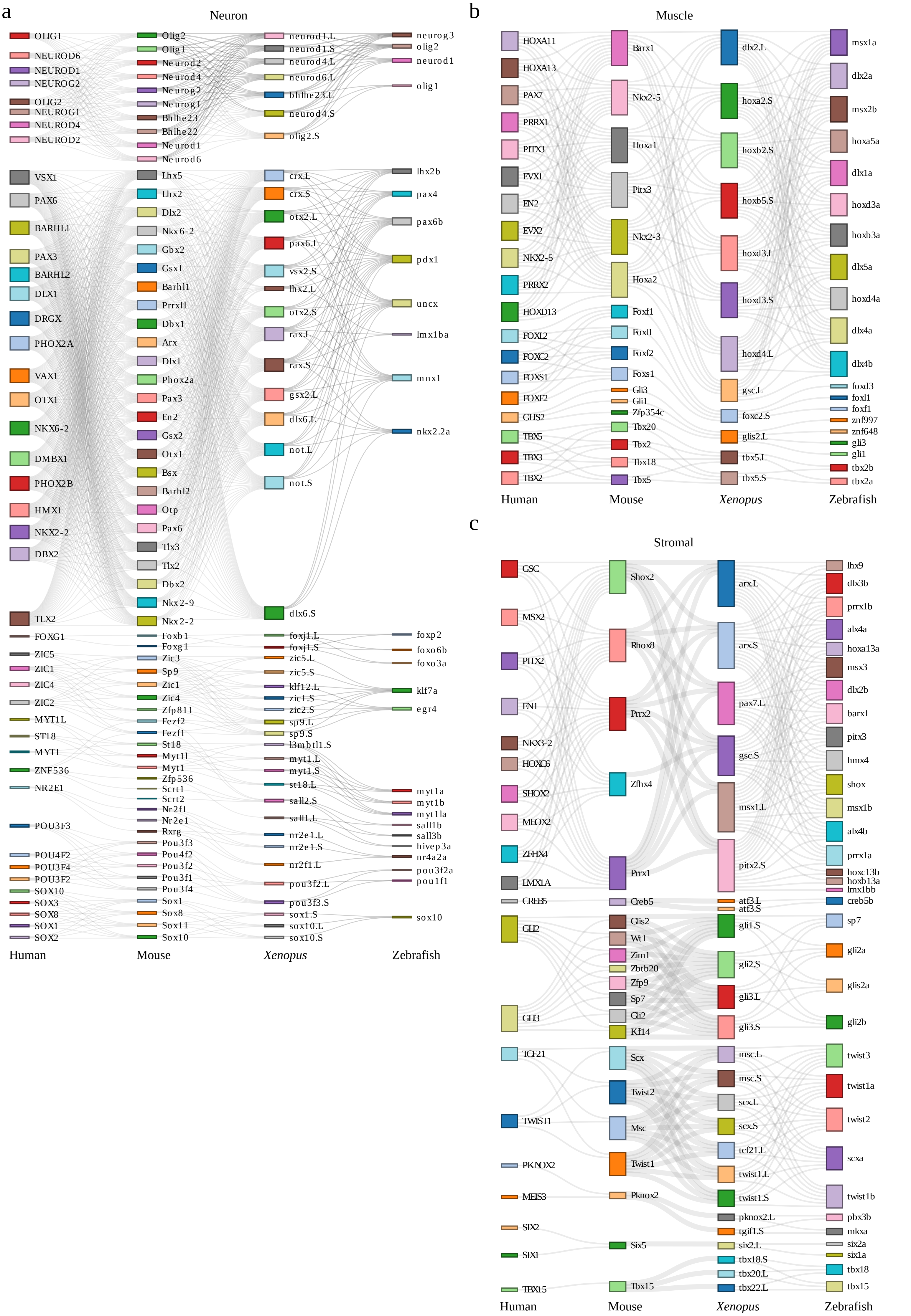

a
Neuron
OLIG1
NEUROD6
NEUROD1
NEUROG2
OLIG2
NEUROG1
NEUROD4
NEUROD2
VSX1
PAX6
BARHL1
PAX3
BARHL2
DLX1
DRGX
PHOX2A
VAX1
OTX1
NKX6-2
DMBX1
PHOX2B
HMX1
NKX2-2
DBX1
TLX2
FOXG1
ZIC5
ZIC1
ZIC4
ZIC2
MYT1L
ST18
MYT1
ZNF536
NR2E1
POU3F3
POU4F2
POU3F4
POU3F2
SOX10
SOX3
SOX8
SOX1
SOX2
Olig2
Olig1
Neurod2
Neurod4
Neurog2
Neurog1
Bhlhe23
Bhlhe22
Neurod1
Neurod6
Lhx5
Lhx2
Dlx2
Nkx6-2
Gbx2
Gsx1
Barhl1
Prrxl1
Dbx1
Arx
Dlx1
Phox2a
Pax3
En2
Gsx2
Otx1
Bsx
Barhl2
Otp
Pax6
Tlx3
Tlx2
Dbx2
Nkx2-9
Nkx2-2
Foxb1
Foxg1
Zic3
Sp9
Zic1
Zic4
Zfp811
Fezf2
Fezf1
St18
Myt1l
Myt1
Zfp536
Scrt1
Scrt2
Nr2f1
Nr2e1
Rxrg
Pou3f3
Pou4f2
Pou3f2
Pou3f1
Pou3f4
Sox1
Sox8
Sox11
Sox10
neurod1.L
neurod1.S
neurod4.L
neurod6.L
bhlhe23.L
neurod4.S
olig2.S
crx.L
crx.S
otx2.L
pax6.L
vsx2.S
lhx2.L
otx2.S
rax.L
rax.S
gsx2.L
dlx6.L
not.L
not.S
dlx6.S
foxj1.L
foxj1.S
zic5.L
zic5.S
klf12.L
zic1.S
zic2.S
sp9.L
sp9.S
l3mbtl1.S
myt1.L
myt1.S
st18.L
sall2.S
sall1.L
nr2e1.L
nr2e1.S
nr2f1.L
pou3f2.L
pou3f3.S
sox1.S
sox10.L
sox10.S
neurog3
olig2
neurod1
olig1
lhx2b
pax4
pax6b
pdx1
uncx
lmx1ba
mnx1
nkx2.2a
foxp2
foxo6b
foxo3a
klf7a
egr4
myt1a
myt1b
myt1la
sall1b
sall3b
hivep3a
nr4a2a
pou3f2a
pou1f1
sox10
Human
Mouse
Xenopus
Zebrafish
b
Muscle
HOXA11
HOXA13
PAX7
PRRX1
PITX3
EVX1
EN2
EVX2
NKX2-5
PRRX2
HOXD13
FOXL2
FOXC2
FOXS1
FOXF2
GLIS2
TBX5
TBX3
TBX2
Barx1
Nkx2-5
Hoxa1
Pitx3
Nkx2-3
Hoxa2
Foxf1
Foxl1
Foxf2
Foxs1
Gli3
Gli1
Zfp354c
Tbx20
Tbx2
Tbx18
Tbx5
dlx2.L
hoxa2.S
hoxb2.S
hoxb5.S
hoxd3.L
hoxd3.S
hoxd4.L
gsc.L
foxc2.S
glis2.L
tbx5.L
tbx5.S
msx1a
dlx2a
msx2b
hoxa5a
dlx1a
hoxd3a
hoxb3a
dlx5a
hoxd4a
dlx4a
dlx4b
foxd3
foxl1
foxf1
znf997
znf648
gli3
gli1
tbx2b
tbx2a
Human
Mouse
Xenopus
Zebrafish
c
Stromal
GSC
MSX2
PITX2
EN1
NKX3-2
HOXC6
SHOX2
MEOX2
ZFHX4
LMX1A
CREB5
GLI2
GLI3
TCF21
TWIST1
PKNOX2
MEIS3
SIX2
SIX1
TBX15
Shox2
Rhox8
Prrx2
Zfhx4
Prrx1
Creb5
Glis2
Wt1
Zim1
Zbtb20
Zfp9
Sp7
Gli2
Kf14
Scx
Twist2
Msc
Twist1
Pknox2
Six5
Tbx15
arx.L
arx.S
pax7.L
gsc.S
msx1.L
pitx2.S
atf3.L
atf3.S
gli1.S
gli2.S
gli3.L
gli3.S
msc.L
msc.S
scx.L
scx.S
tcf21.L
twist1.L
twist1.S
pknox2.L
tgif1.S
six2.L
tbx18.S
tbx20.L
tbx22.L
lhx9
dlx3b
prrx1b
alx4a
hoxa13a
msx3
dlx2b
barx1
pitx3
hmx4
shox
msx1b
alx4b
prrx1a
hoxc13b
hoxb13a
lmx1bb
creb5b
sp7
gli2a
glis2a
gli2b
twist3
twist1a
twist2
scxa
twist1b
pbx3b
mkxa
six2a
six1a
tbx18
tbx15
Human
Mouse
Xenopus
Zebrafish

Supplementary Figure 8. The common lineage-specific TFs for each lineage. Sankey plot showing common lineage-specific TFs in neuron (a), muscle (b) and stromal (c). Different TFs in each species are marked in different color boxes. Homologous TFs between species obtained from SAMap are connected by lines.

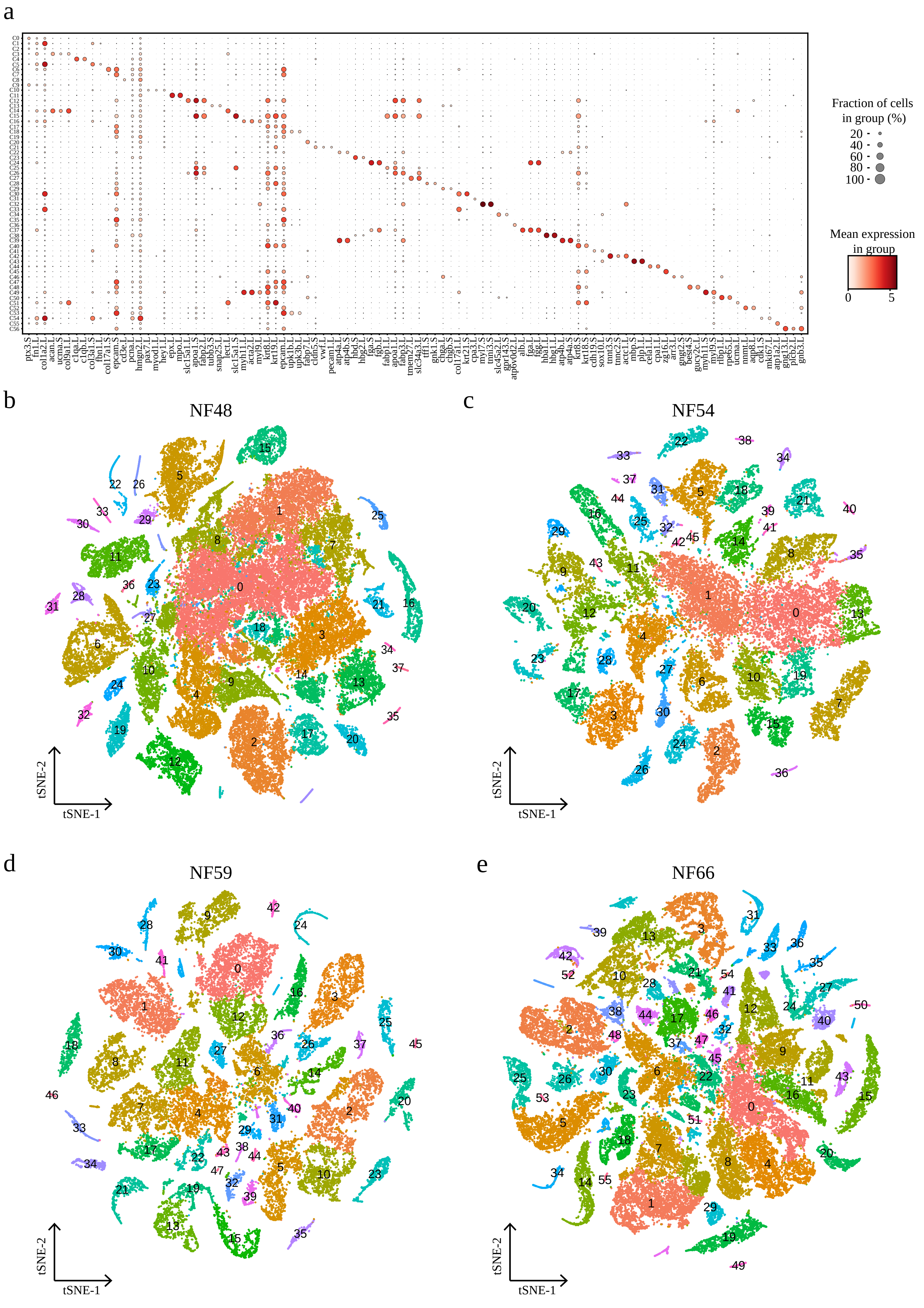
a
ptx3.S fn1.L col1a2.L acan.L ucma.S col9a1.L c1qa.L c1qb.L col3a1.S fbn1.L col17a1.S epcam.S cd3e.L pcna.L hmgn2.L pax7.L myod1.L hey1.L epx.L mpo.L slc15a1.L apoa1.S fabp2.L tubb3.S snap25.L lect.L slc15a1.S myh11.L acta2.L myl9.L krt8.L krt19.L epcam.L upk1b.L upk3b.L fabp7.L cldn5.S vwf.L pecam1.L atp4a.L atp4b.S hbd.S hbg2.L fga.S fgb.L fabp1.L apoa1.L fabp3.L tmem27.L slc34a3.L tff1.S gnk1.S chga.L chgb.S col17a1.L krt21.S cpa3.L myl7.S tnni3.S slc45a2.L gpr143.S atp6v0d2.L alb.L fga.L fgg.L hba1.L hbg1.L atp4b.L atp4a.S krt8.S krt18.S cdh19.S sox10.L tnnt3.S tnnc2.S actc1.L mbp.S plp1.L cela1.L cpa1.L zg16.L arr3.L gngt2.S best4.S gucy2c.L myh11.S myl9.S rlbp1.L rpe65.L ucma.L nnmt.L aqp8.L cdk1.S mki67.L atp1a2.L gng13.L plcb2.L gnb3.L
Fraction of cells in group (%)
20 40 60 80 100
Mean expression in group
0 5
b
NF48
c
NF54
d
NF59
e
NF66
tSNE-1
tSNE-2

Supplementary Figure 9. Details of the constructed larval *Xenopus* cell atlas during metamorphosis. a, Dot plot showing representative gene expression in each cluster of larval *Xenopus* during metamorphosis. The size of the dot encodes the percentage of cells within a cell type, and the color encodes the average expression level. b-e, t-SNE analysis of four tadpole stages collected to represent larval *Xenopus*. Cells are colored by cell-type cluster.

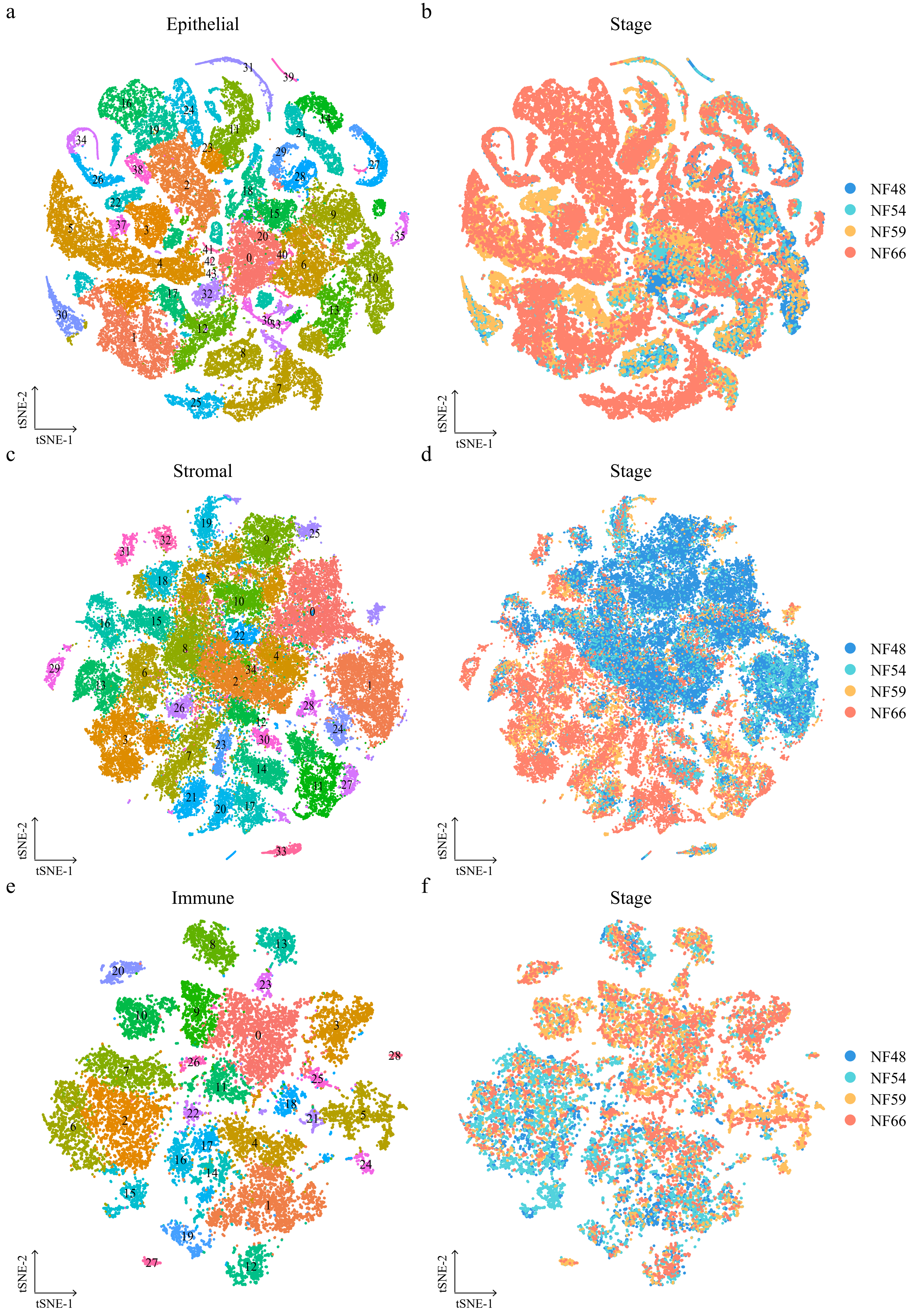
a
Epithelial
b
Stage
NF48
NF54
NF59
NF66
c
Stromal
d
Stage
NF48
NF54
NF59
NF66
e
Immune
f
Stage
NF48
NF54
NF59
NF66
tSNE-1
tSNE-2

Supplementary Figure 10. Cellular heterogeneity of larval *Xenopus* cell lineages. t-SNE maps of single-cell data from larval epithelial (a), stromal (c) and immune (e) during metamorphosis. Cells are colored by cell-type cluster; t-SNE maps of single-cell data from larval epithelial (b), stromal (d) and immune (f) during metamorphosis. Cells are colored by stage.

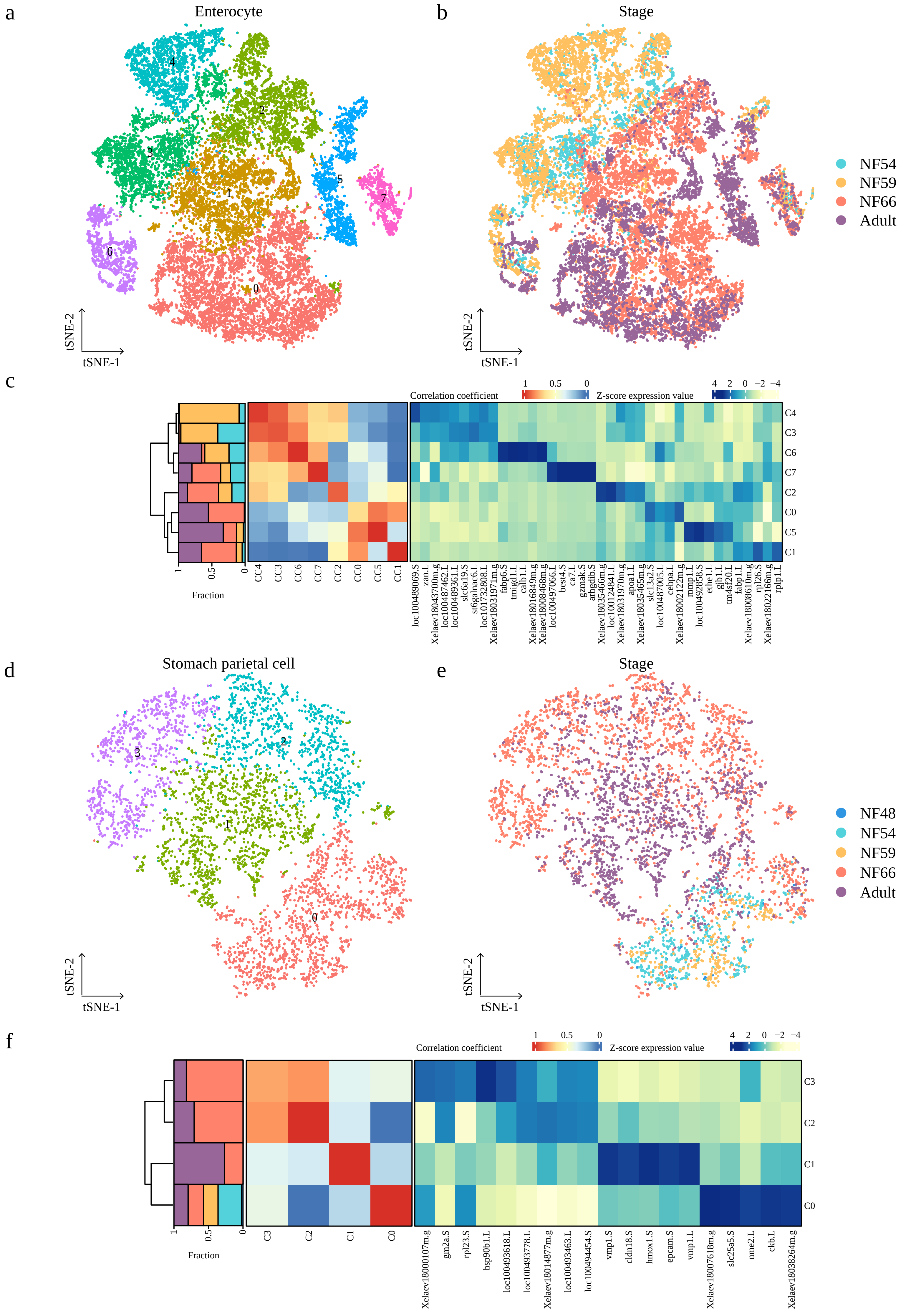
a
Enterocyte
4
2
3
1
5
7
6
0
tSNE-2
tSNE-1
b
Stage
NF54
NF59
NF66
Adult
tSNE-2
tSNE-1
c
Correlation coefficient
1
0.5
0
Z-score expression value
4
2
0
−2
−4
C4
C3
C6
C7
C2
C0
C5
C1
1
0.5
0
Fraction
CC4
CC3
CC6
CC7
CC2
CC0
CC5
CC1
loc100489069.S
zan.L
Xelaev18043700m.g
loc100487462.L
loc100489361.L
slc6a19.S
st6galnac6.L
loc101732808.L
Xelaev18031971m.g
fabp6.S
tmigd1.L
calb1.L
Xelaev18016849m.g
Xelaev18008468m.g
loc100497066.L
best4.S
ca7.L
gzmak.S
arhgdib.S
Xelaev18035466m.g
loc100124841.L
Xelaev18031970m.g
apoa1.L
Xelaev18035465m.g
slc13a2.S
loc100487005.L
cebpa.L
Xelaev18002122m.g
mmp1.L
loc100492858.S
ethe1.L
gjb1.L
tm4sf20.L
fabp1.L
Xelaev18008610m.g
rpl26.S
Xelaev18022166m.g
rplp1.L
d
Stomach parietal cell
3
2
1
0
tSNE-2
tSNE-1
e
Stage
NF48
NF54
NF59
NF66
Adult
tSNE-2
tSNE-1
f
Correlation coefficient
1
0.5
0
Z-score expression value
4
2
0
−2
−4
C3
C2
C1
C0
1
0.5
0
Fraction
C3
C2
C1
C0
Xelaev18000107m.g
gm2a.S
rpl23.S
hsp90b1.L
loc100493618.L
loc100493778.L
Xelaev18014877m.g
loc100493463.L
loc100494454.S
vmp1.S
cldn18.S
hmox1.S
epcam.S
vmp1.L
Xelaev18007618m.g
slc25a5.S
nme2.L
ckb.L
Xelaev18038264m.g

Supplementary Figure 11. The correlation between larval and adult cell types. t-SNE maps of single-cell data from larval and adult enterocytes (a) and stomach parietal cells (d). Cells are colored by cell-type cluster; t-SNE maps of single-cell data from larval and adult enterocytes (b) and stomach parietal cells (e). Cells are colored by stage. Heatmap showing representative gene expression and correlation of larval and adult enterocytes (c) and stomach parietal cells (f). AUROC scores were used to measure the similarity of cell types: red, high correlation; blue and yellow, low correlation, based on the Spearman correlation. The stacked bar plots illustrate the proportion of clusters. Stage is marked by different colors same as b and d, respectively.

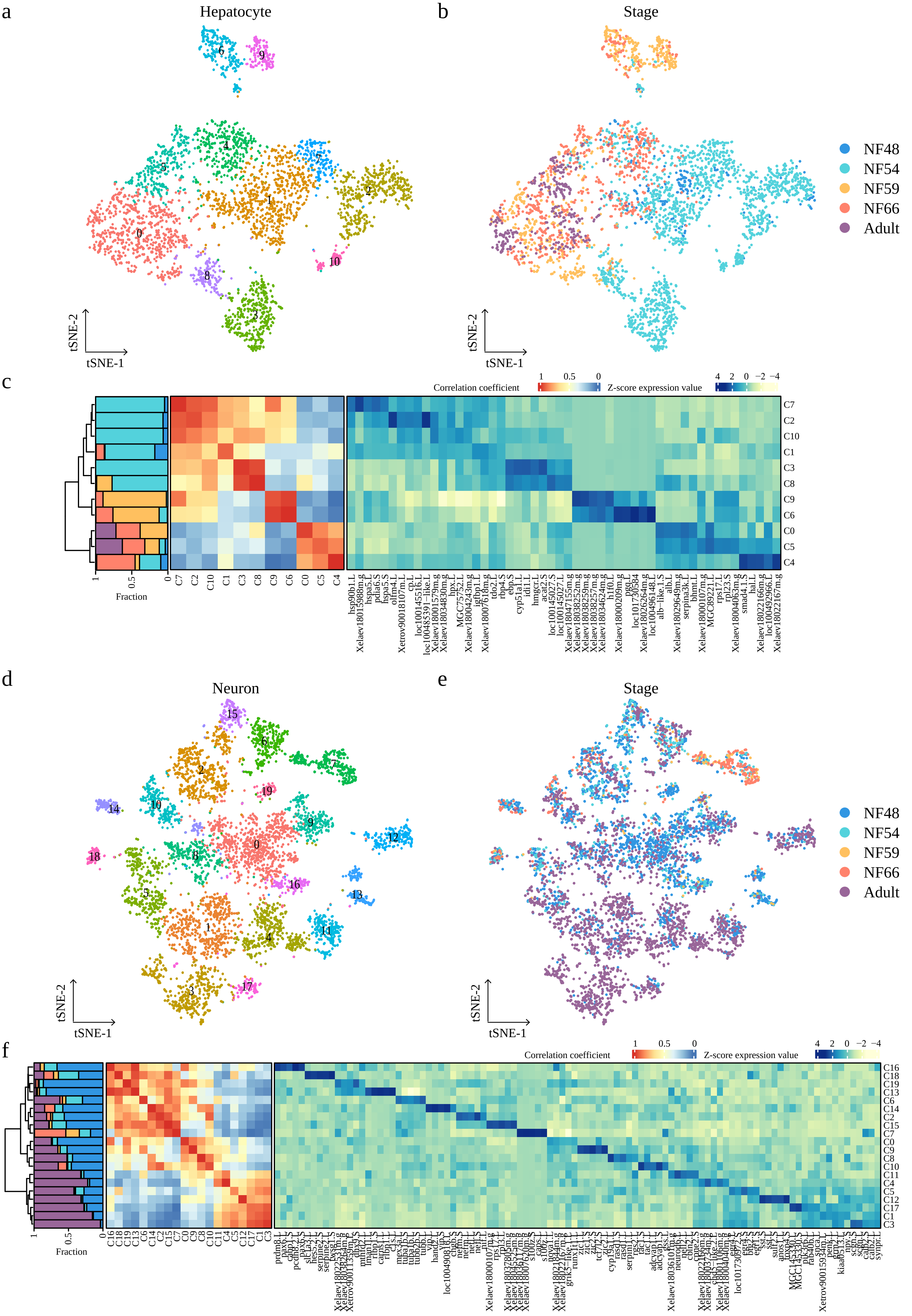

a
Hepatocyte
tSNE-2
tSNE-1
b
Stage
NF48
NF54
NF59
NF66
Adult
c
Correlation coefficient
1
0.5
0
Z-score expression value
4
2
0
−2
−4
C7
C2
C10
C1
C3
C8
C9
C6
C0
C5
C4
Fraction
d
Neuron
e
Stage
f
C16
C18
C19
C13
C6
C14
C2
C15
C7
C0
C9
C8
C10
C11
C4
C5
C12
C17
C1
C3

Supplementary Figure 12. The correlation between larval and adult cell types. t-SNE maps of single-cell data from larval and adult hepatocytes (a) and neurons (d). Cells are colored by cell-type cluster; t-SNE maps of single-cell data from larval and adult hepatocytes (b) and neurons (e). Cells are colored by stage. Heatmap showing representative gene expression and correlation of larval and adult hepatocytes (c) and neurons (f). AUROC scores were used to measure the similarity of cell types: red, high correlation; blue and yellow, low correlation, based on the Spearman correlation. The stacked bar plots illustrate the proportion of clusters. Stage is marked by different colors same as b and d, respectively.
