# Supplementary material for: Cell landscape of larval and adult Xenopus laevis at single-cell resolution
Source: Nat Commun. 2022 Jul 25;13:4306. doi: 10.1038/s41467-022-31949-2 (PMC9314398; doi:10.1038/s41467-022-31949-2)
Supplement: Supplementary file 2 — Description of additional Supplementary File [file 41467_2022_31949_MOESM2_ESM.pdf]

Descriptions of Additional Supplementary Data files

Supplementary Dataset 1. Tissue dissociation information and Xenopus/tadpole cell landscape.

Supplementary Dataset 2. Marker genes for each tissue/stage.

Supplementary Dataset 3. Homologous gene information across four species

Supplementary Dataset 4. Marker genes for epithelial, stromal and immune.

Supplementary Dataset 5. Marker genes for enterocyte, stomach parietal cell, neuron and hepatocyte.

Supplementary Dataset 6. Driver TFs for each cell lineage during metamorphosis.

Supplementary Dataset 7. Genes selected for each module.

Supplementary Dataset 8. GO term for each module.

Supplementary Dataset 9. Oligonucleotide sequences in our manuscript.
