# Supplementary material for: Cell landscape of larval and adult Xenopus laevis at single-cell resolution
Source: Nat Commun. 2022 Jul 25;13:4306. doi: 10.1038/s41467-022-31949-2 (PMC9314398; doi:10.1038/s41467-022-31949-2)
Supplement: Supplementary file 12 — Reporting Summary [file 41467_2022_31949_MOESM12_ESM.pdf]

Corresponding author(s): Xiaoping Han, Guoji Guo, Wanhua Shen.

Last updated by author(s): Jun 21, 2022

Reporting Summary

Nature Portfolio wishes to improve the reproducibility of the work that we publish. This form provides structure for consistency and transparency in reporting. For further information on Nature Portfolio policies, see our Editorial Policies and the Editorial Policy Checklist.

Statistics

For all statistical analyses, confirm that the following items are present in the figure legend, table legend, main text, or Methods section.

n/a	Confirmed	
☐	☒	The exact sample size (*n*) for each experimental group/condition, given as a discrete number and unit of measurement
☐	☒	A statement on whether measurements were taken from distinct samples or whether the same sample was measured repeatedly
☐	☒	The statistical test(s) used AND whether they are one- or two-sided<br>*Only common tests should be described solely by name; describe more complex techniques in the Methods section.*
☒	☐	A description of all covariates tested
☒	☐	A description of any assumptions or corrections, such as tests of normality and adjustment for multiple comparisons
☐	☒	A full description of the statistical parameters including central tendency (e.g. means) or other basic estimates (e.g. regression coefficient) AND variation (e.g. standard deviation) or associated estimates of uncertainty (e.g. confidence intervals)
☐	☒	For null hypothesis testing, the test statistic (e.g. F, t, r) with confidence intervals, effect sizes, degrees of freedom and P value noted<br>*Give P values as exact values whenever suitable.*
☒	☐	For Bayesian analysis, information on the choice of priors and Markov chain Monte Carlo settings
☒	☐	For hierarchical and complex designs, identification of the appropriate level for tests and full reporting of outcomes
☐	☒	Estimates of effect sizes (e.g. Cohen's *d*, Pearson's *r*), indicating how they were calculated

Our web collection on statistics for biologists contains articles on many of the points above.

Software and code

Policy information about availability of computer code

Data collection	No software was used for data collection.
Data analysis	splitBarcode, Version 0.1.6 (available at http://github.com/MGI-tech-bioinformatics/splitBarcode)<br>Dropseq_tools, Version 1.12 (available at http://mccarrolllab.com/dropseq/)<br>Seurat, Version 3 (available at http://satijalab.org/seurat/)<br>STAR, Version 2.5.2 (available at http://github.com/alexdobin/STAR)<br>Scanpy, Version 1.6.0 (available at http://github.com/theislab/scanpy/)<br>MetaNeighbor, Version n/a (available at http://github.com/maggiecrow/MetaNeighbor)<br>R, Version 3.6.3 (available at https://www.r-project.org/)<br>The detail code can be accessed on github (https://github.com/ggjlab/XCL)

For manuscripts utilizing custom algorithms or software that are central to the research but not yet described in published literature, software must be made available to editors and reviewers. We strongly encourage code deposition in a community repository (e.g. GitHub). See the Nature Portfolio guidelines for submitting code & software for further information.

Data

Policy information about availability of data

All manuscripts must include a data availability statement. This statement should provide the following information, where applicable:

- Accession codes, unique identifiers, or web links for publicly available datasets
- A description of any restrictions on data availability
- For clinical datasets or third party data, please ensure that the statement adheres to our policy

scRNA-seq data generated by this work have been deposited in the NCBI Gene Expression Omnibus database (https://www.ncbi.nlm.nih.gov/geo/) and are accessible through the following accession number GEO: GSE195790. scRNA-seq processed data have been deposited on Figshare (https://figshare.com/s/08d6520c77f37af639ce). scRNA-seq data can also be accessed on the website (http://bis.zju.edu.cn/XCL/). The detail code can be accessed on github (https://github.com/ggjlab/XCL).

Field-specific reporting

Please select the one below that is the best fit for your research. If you are not sure, read the appropriate sections before making your selection.

☒ Life sciences ☐ Behavioural & social sciences ☐ Ecological, evolutionary & environmental sciences

For a reference copy of the document with all sections, see nature.com/documents/nr-reporting-summary-flat.pdf

Life sciences study design

All studies must disclose on these points even when the disclosure is negative.

Sample size	We dissociated the whole tissue and whole-body tadpole to generate single cell suspension. 303,442 adult Xenopus laevis single cells and 197,916 tadpole single cells were analyzed in total. A total of 17 adult Xenopus laevis tissues and tadpoles at 4 different stages were analyzed.
Data exclusions	Data of the whole tissue and whole-body tadpole points with fewer than 500 UMI were excluded. The detected transcript from a single cell under average sequencing depth (3500 reads/cell) should be more than 500 UMI. Cell barcodes with less than 500 UMI usually correspond to empty beads exposed to free RNA during cell lysis, RNA capture and washing steps.
Replication	1-3 replications were done for different tissues when samples were available. 1 replication was done for tadpoles at different stages . The results of major cell type clusters are reproducible.
Randomization	Different single cells were randomly captured before analysis. All Xenopus were of d/d genetic background. Animal samples were not randomized. Integration methods in Scanpy and Seurat controlled co-variates and clustered single-cell RNA sequencing data across different replicates.
Blinding	We are blinded to analyzed cell types before single cell analyses.

Reporting for specific materials, systems and methods

We require information from authors about some types of materials, experimental systems and methods used in many studies. Here, indicate whether each material, system or method listed is relevant to your study. If you are not sure if a list item applies to your research, read the appropriate section before selecting a response.

Materials & experimental systems

n/a	Involved in the study
☒	☐ Antibodies
☒	☐ Eukaryotic cell lines
☒	☐ Palaeontology and archaeology
☐	☒ Animals and other organisms
☒	☐ Human research participants
☒	☐ Clinical data
☒	☐ Dual use research of concern

Methods

n/a	Involved in the study
☒	☐ ChIP-seq
☒	☐ Flow cytometry
☒	☐ MRI-based neuroimaging

Animals and other organisms

Policy information about studies involving animals; ARRIVE guidelines recommended for reporting animal research

Laboratory animals	Adult wide-type Xenopus laevis tissues were collected from 1 year old male and female animals. Tadpoles (random sex) at different

Laboratory animals	stages were collected from Xenopus laevis at NF48, NF56, NF59 and NF66. All animals housed in a 22 °C incubator with 12 h light /12 h dark cycle.
Wild animals	The study did not involve wild animals.
Field-collected samples	No field collected samples were used in the study
Ethics oversight	All animal protocols in this study were approved by the Ethics Committee of the Zhejiang University Laboratory Animal Center (Lot number: 20201049 and ZJU20220195).

Note that full information on the approval of the study protocol must also be provided in the manuscript.
